# Attempts to investigate what words trivia experts focus on in quiz questions: Human-AI comparison through “LLM-as-a-judge” approach

**DOI:** 10.1371/journal.pone.0358332

**Published:** 2026-09-22

**Authors:** Masaru Shirasuna, Yuto Yoshida

**Affiliations:** Faculty of Informatics, Shizuoka University, Hamamatsu-shi, Shizuoka, Japan; Samsun University: Samsun Universitesi, TÜRKIYE

## Abstract

Computational capacity and knowledge of humans are more limited than those of large language models (LLMs). However, in buzzer quizzes, human trivia experts can often identify the correct answer even from insufficient information such as only a few words. Investigating how they can make fast and accurate judgments is expected to highlight new characteristics of human intelligence, but little is known about that. In this exploratory and case-based analysis, we predicted that trivia experts and LLMs would differ in which words/phrases in a question are important for identifying the answer, and compared experts’ performance with LLMs’ performance in Japanese trivia questions through an LLM-as-a-judge approach: We regarded LLMs as evaluators and then used their outputs as comparative tools for experts’ evaluations. First, we constructed a quiz question processing system that tokenized question texts based on morphological analysis and then numerically evaluated the importance of each token using GPT-4o/GPT-4.1. Then, we conducted a behavioral experiment wherein actual trivia experts were asked to numerically evaluate the importance of each token, just as LLMs had performed. As a result, trivia experts treated a variety of words/phrases as important, even if each word/phrase did not appear to be strongly associated with the answer. Their evaluation scores tended to accumulate faster than those of the LLMs. This may indicate that trivia experts can make inductive inferences faster (e.g., finding a common concept even from few items). More advanced question-answering systems may be designed by applying trivia experts’ cognitive processes to LLMs’ information processing, and our findings may provide a scaffolding toward such goals.

## 1. Introduction

Consider the following trivia question:

*The nutrients essential to the human body, known as the “three major nutrients,” are fats, proteins, and what?*

The answer is “Carbohydrates”. Many people (and computers) can probably answer this question. However, at what point did you figure out the answer?

Next, consider the following incomplete sentence

*The nutrients essential to the human body, known as the “three major nutrients,” are fats, p…*

What about this case? Although the question text is incomplete, some people will still know the answer. This is because they can infer the “p” as in “protein” and thus the remaining item must be “Carbohydrates”. In this question text, “p” (an initial letter of “protein”) is a critical key point to uniquely identify the correct answer, and humans are sometimes highly sensitive to such a point.

### 1.1 Fast and accurate judgments under uncertainty with limited cognitive resources

Suppose a trivia question like “What/Who/Where/When is this?”. We are sometimes required to make judgments under uncertainty in daily life: Only insufficient information or fragmented words/phrases are available because, for example, the questioner has forgotten the details.

Recent artificial intelligence (AI) systems, specifically large language models (LLMs), have much more computational capacity and information processing ability than humans [1]. They can sometimes make more sophisticated output than humans if sufficient information (e.g., rich and detailed prompts; enough cues to uniquely identify the answer) is given as input.

However, humans sometimes perform well in terms of being able to make judgments even from limited information [2–4]. In answering a question, humans may be able to capture an important letter, word, or phrase more sensitively (e.g., “p” as “protein” in the above “Carbohydrates” question) than AIs do. In other words, it is expected that the words/phrases that are regarded as important in a trivia question will differ between humans (especially trivia experts) and AIs. Clarifying such differences will provide further understanding of how humans make fast and accurate judgments under cognitive constraints and uncertainty. Furthermore, if we can obtain empirical evidence that humans can identify the answer faster than AI in trivia questions, this finding may potentially be applied to more advanced AI design in the future (e.g., by tuning LLMs to behave more like humans; see also [5]).

### 1.2 Trivia quiz questions and cognitive processes of trivia experts

As a material to investigate human intelligence for trivia questions under limited information, we focus on trivia quizzes. Competitive trivia is widely enjoyed around the world, in such forms as primetime TV programs, quiz-game apps, web-media content, and gamified education. A buzzer quiz, known as “hayaoshi quiz” in Japanese, is a form of knowledge-based competition and one of the best-known popular entertainments especially in Japan [6–8]. In a buzzer quiz, a trivia question is provided in a question-answer format (i.e., a question text is read by a questioner; e.g., “The ruins of Ilios, Cappadocia, and the Topkapi Palace are famous tourist spots in this country. Which country is it?” --- Answer: Turkey). Players are required to make a correct judgment faster than other competitors, by pushing a button even at the middle of a question text. That is, even if they would obtain sufficient information for the answer by hearing the whole question text, they sometimes have to reach an answer from insufficient information before any other player pushes their button.

To answer questions faster and more accurately than their opponents in buzzer-quiz situations, quiz players often study trivia questions considering which words/phrases in a sentence will be important for identifying the correct answer [9]. In actual competitive buzzer quizzes, experts tend to sensitively capture a critical keyword (i.e., push a button immediately when the keyword is heard), which enables them to identify the correct answer as fast and as accurately as possible even from insufficient information [6,10]. For example, in the above “Turkey” question, a quiz player can push a button at the time s/he hears “The ruins of Ilios, Cappadocia,…” and infer the answer as Turkey. In this situation, s/he would consider “Cappadocia … should be associated with Turkey!”, and thus it is assumed that s/he regards “Cappadocia” as a highly important cue. Thus, it is considered that trivia experts generally know which and to what extent words/phrases would be associated with the answer of trivia quiz questions (see also [11]).

Compared to LLMs, what are the characteristics of how trivia experts consider quizzes? AI clearly has advanced computational capacity that enables it to handle vast amounts of data [12,13]. However, trivia experts can make fast and accurate judgments even from fragmented information or uncertainty (see also [14]). What words/phrases do trivia experts consider to be important cues in leading them to the correct answer?

### 1.3 “LLM-as-a-judge” approach

To investigate human trivia experts’ cognitive processes compared to AI performance, this study used the “LLM-as-a-judge” approach [15]. LLM-as-a-judge is an approach in which researchers treat an LLM as “a judge” or “an evaluator” for complex tasks. Given recent LLMs’ ability to process diverse data types and to provide scalable, cost-effective, and consistent assessments, they can be used as a useful alternative to traditional expert-driven evaluations [16]. Previous studies have shown that by using appropriately trained LLMs, LLM-as-a-judge is an approach to approximate human preferences (which are sometimes expensive to obtain) in many tasks such as a multi-turn question set [17] and multimodal models [18].

In this study, we attempt to discuss human-specific intelligence in answering quiz questions, through human-AI comparison (e.g.[19–22]). Specifically, we construct a quiz question processing system that requires LLMs to quantitatively evaluate the extent of importance of words/phrases to identify the correct answer in a question text. We then use the LLMs’ evaluation as a kind of comparative tool. To apply such an approach, LLMs need to be tuned for answering quiz questions. Thus, before a behavioral experiment, we implement the system to return valid and plausible evaluations for each word (for details, see the Methods section).

### 1.4 Study outline

To clarify the characteristics of human judgment processes under uncertainty (focusing on trivia experts in buzzer quizzes), we compared trivia experts’ performance with LLMs’ performance in quiz questions through an LLM-as-a-judge approach. First, as a theoretical study, we constructed a quiz question processing system consisting of two modules: One module segmented a question text into words/phrases (i.e., tokenization) based on morphological analysis; and the other used LLMs to score numerically how important each word/phrase was for identifying the correct answer. Then, as a behavioral experiment for humans (trivia experts), we conducted the same tasks as the LLMs performed in the theoretical study; that is, we asked actual trivia experts to evaluate the importance of each word/phrase in questions. By comparing LLMs’ and experts’ evaluations, we discuss the similarities and differences in the words/phrases they focus on in quiz questions.

Our focus is characteristics of trivia experts’ cognition, rather than improving AI systems or establishing new standards for LLM-as-a-judge approaches. We use LLMs as comparative analytical (rather than as normative) tools. The aim is to find some similarities/differences between humans and LLMs through comparison of their performance in quiz questions. Note that we position this study as an exploratory, case-based study. This is because, as described below, the sample size in the behavioral experiment was small. The findings obtained in this study should be regarded as non-definitive evidence (e.g., clarifying not general human cognitive processes but expert-specific tendencies).

## 2. Methods

In this study, we adopted 2,480 quiz questions, which were used in actual, traditional and large-scale Japanese annual buzzer quiz competitions, named abc the 11^th^ and 12^th^ (held in March 2013 and March 2014, respectively). These questions covered various genres such as history, science, and sports, etc. All quiz questions were in a one-question-one-answer format and consisted of approximately 60 Japanese characters. For example:

The ruins of Ilios, Cappadocia, and the Topkapi Palace are famous tourist spots in this country. Which country is it? — answer: Turkey

(Japanese original text: イリオス遺跡、カッパドキア、トプカプ宮殿などの観光地がある国といえばどこでしょう? — 正答: トルコ)

### 2.1 Theoretical study: Implementing “quiz question processing system”

**Fig 1:**
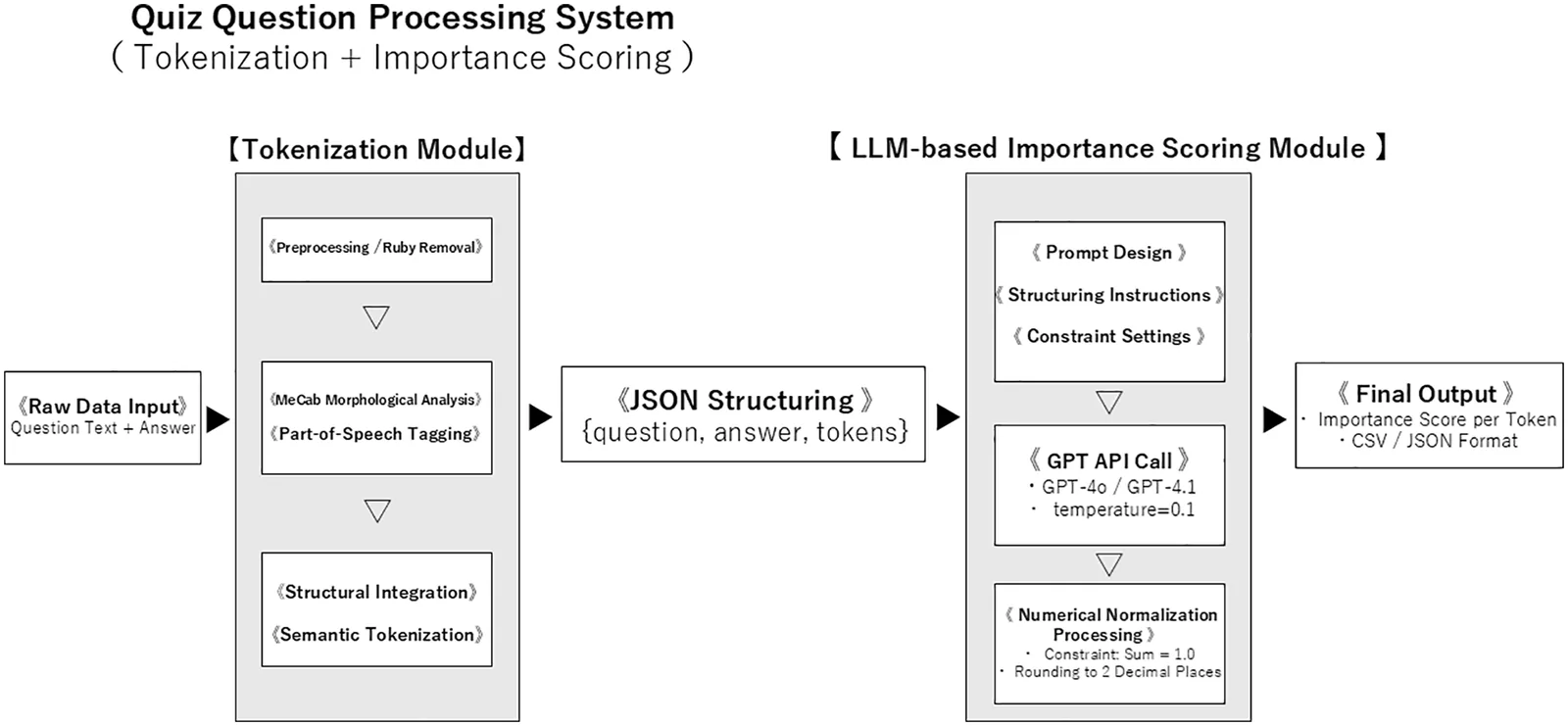
Schematic of constructing the quiz question processing system. The system had two modules: The tokenization module segmented each quiz question into tokens (words or phrases), and the importance-scoring module numerically evaluated the importance of each token for leading the correct answer.

We implemented a “quiz question processing system”, which quantified the extent to which each token (i.e., word or phrase) in a quiz question would contribute to identifying the correct answer. As shown in Fig 1, this system consisted of two modules: Tokenization module, and an LLM-based importance-scoring module. The tokenization module segmented a question text into words/phrases through morphological analyses. Then, the LLM-based importance-scoring module numerically evaluated the extent of importance of each word/phrase for leading the answer based on LLMs (LLM-as-a-judge approach). In the main text, we report only a brief outline of the system pipeline. For details, see Supporting information 01 in S1 File.

#### 2.1.1 Tokenization module.

We first segmented each question text into words/phrases (i.e., tokenizing). The input data was a structure of buzzer-quiz question scripts, and the output data was provided as a JSON object of a pair of question texts and answers. The Python code for tokenizing is available at https://osf.io/hvfsn/overview.

Next, each question was analyzed morphologically by using a Japanese open-source engine, MeCab [23]. We extracted part-of-speech tags at a lexical level. We then merged some words/phrases on semantic and syntactic grounds (e.g., a sequence of part-of-speeches). For all 2,480 quiz questions and their tokenization results, see https://osf.io/hvfsn/overview.

#### 2.1.2 LLM-based importance-scoring module.

Next, we asked the LLMs to evaluate how much each token (tokenized by the previous module) could serve as an important cue for identifying the correct answer. The general procedure of LLMs’ evaluation was to assign each token a value ranging from 0.00 (not important at all) to 1.00 (highly important) adjusting the sum of values to 1.00. We used the two widely used LLMs, GPT-4o and GPT-4.1, with identical prompt designs and hyperparameters to ensure comparability of outputs. This module included the following three main processes. Codes and prompts are available at https://github.com/bravebird0914/llm-quiz-attribution.

**- Prompt design**

We provided the following structured instructions to LLMs.

1. Assign values close to 0.00 to tokens providing no cues,
2. Assign values close to 1.00 to highly useful tokens,
3. Adjust the total values of all tokens to 1.00,
4. Output to two decimal places,
5. Return in JSON format.

To ensure consistency in evaluation criteria, we set the following prompt: “You are a trivia expert. Accurately evaluate the importance of the given tokens.” as the LLM’s system role. Hereafter, the numerical values of importance assigned to each token are called “system’s evaluation score”.

**- GPT API call and** ***temperature*** **parameter**

For both GPT-4o and GPT-4.1, we used a method from the OpenAI Python library, client.chat.completions.create(), which allowed us to interact with the Chat Completions API and to generate conversational responses from LLMs. We set the *temperature* parameter = 0.1 (larger values of temperature mean more randomness). We chose the small temperature value of 0.1 because a person’s judgment in buzzer quizzes is generally assumed to be stable. That is, even if a person is presented with a question repeatedly, the person’s evaluations of which word is more important are likely to remain stable [9]. Because our task assessed respondents’ general knowledge rather than creativity, there was no need to assume divergent thinking (represented by a larger temperature). After this process, we obtained attention_weights consisting of an array of tokens and system’s evaluation scores.

**- Numerical normalization processing**

**Fig 2:**
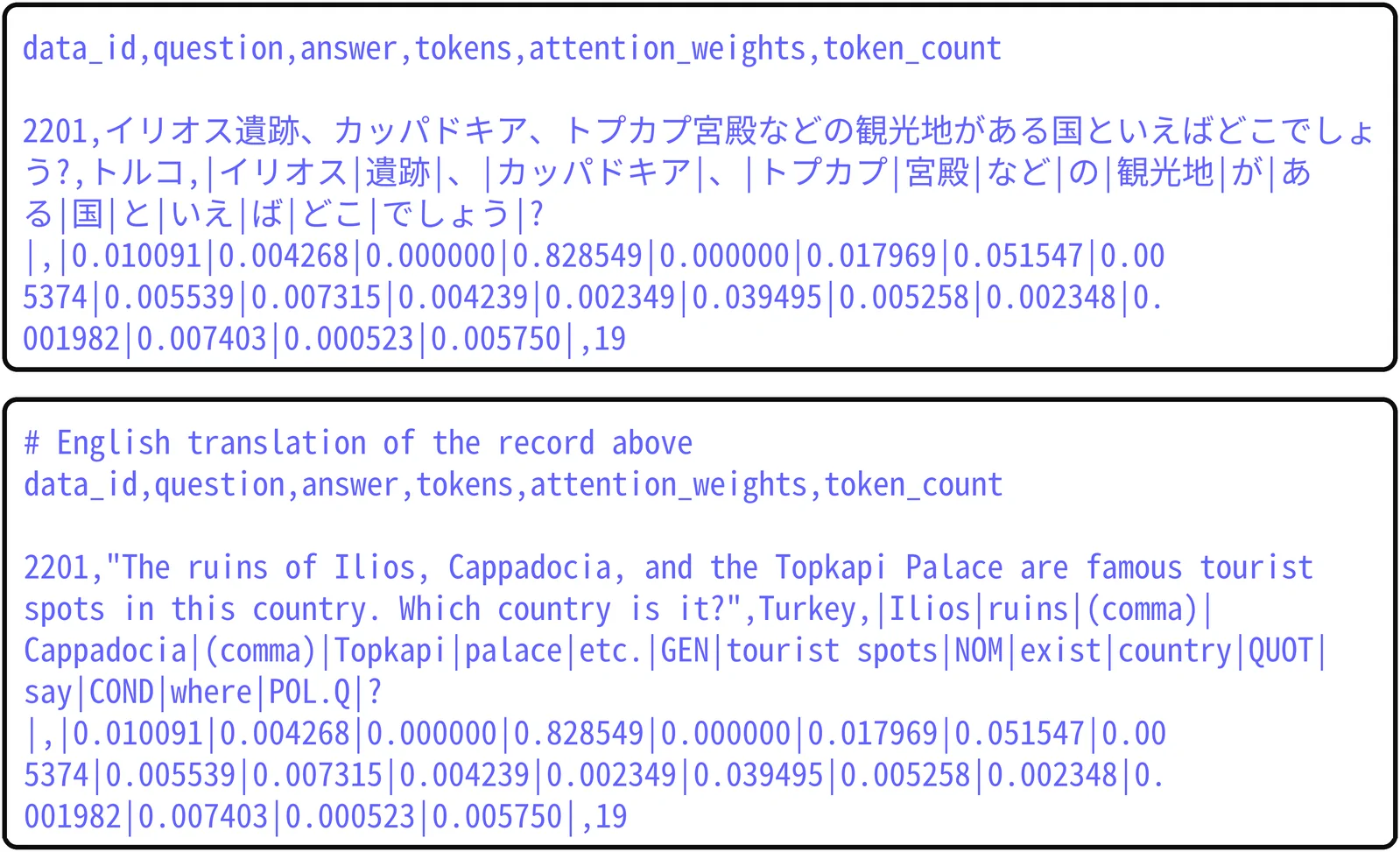
Output example. The system output was provided as a CSV object. Each element denotes the following. “data_id”: question number. “question”: original question text. “answer”: the correct answer. “tokens”: tokens segmented by |. “attention_weights”: system’s evaluation score for each token. “token_count”: the number of tokens in the question. Since the database was written in Japanese, the original analyses were conducted in Japanese (upper). The English translation is provided to aid readers’ understanding (lower; note that Japanese tokens do not correspond exactly one-to-one with their English translations).

The system’s evaluation scores were normalized by rounding and by adjusting the total sum: (i) any negative values were replaced with 0, (ii) each score was scaled by dividing by the total sum to ensure the total sum = 1.00, and (iii) each score was discretized to the second decimal place. Scores of each question were stored as a CSV object with the token sequence and system’s evaluation score sequence separated by | (Fig 2).

As a validity check for the system, the final outputs of tokenization and evaluation scores were briefly scanned by two quiz players (with two and seven years of competitive quiz experience, respectively) throughout the 2,480 questions. Then, both agreed that the output was valid and plausible, with no conspicuously anomalous output. Note that to confirm the robustness of LLMs’ performance, we additionally manipulated the role prompt (Prompt and No-prompt) and temperature parameter (0.1 and system default). Specifically, we tried to prompt LLMs without “You are a trivia expert” to confirm that LLMs’ performance was driven by the models’ own inference ability rather than by the role prompt. We also varied the temperature to the default value of 0.8, which represents more randomness of LLMs’ output. Regardless of these manipulations, LLMs’ performance remained almost unchanged (see Supporting information 02 in S1 File).

We report the system’s evaluation scores together with the results of the subsequent behavioral experiment. Because our main interest was to compare trivia experts’ and LLMs’ evaluations of the importance of each word/phrase, we focus exclusively on the questions used in the behavioral experiment.

### 2.2 Behavioral experiment: Investigating to what extent humans evaluate each word/phrase as important

To clarify similarities and differences between humans and LLMs in solving quizzes, we conducted a behavioral experiment on trivia experts using the same procedures as those applied to LLMs (in LLM-based importance-scoring module). We explored how trivia experts captured each word/phrase in a question text; that is, how their evaluations of importance would differ from LLMs’ evaluations.

#### 2.2.1 Participants.

Eleven trivia experts in buzzer quizzes were recruited (*n*_female_ = 1; *M*_age_ = 26.8, *SD*_age_ = 7.99; from August 11, 2025, to August 29, 2025). They were all Japanese and their first language was Japanese. All had several years of experience participating in actual quiz competitions (quiz experience: *M*_years_ = 7.30; *SD*_years_ = 6.06). They were recruited personally by one of the authors, who was also once a competitive quiz player. The sample size was determined based on a previous study [8]. Prior to the experimental task, all participants provided informed consent online (only those who agreed to participate proceeded to the subsequent task). The experimental protocols in this study conformed to the Declaration of Helsinki and were approved by the Ethics Review Committee for Experimental Research at *Anonymized* (number: 25−16).

#### 2.2.2 Materials.

According to [6], quiz questions can be categorized into several task structures in terms of sentence format. We selected 18 trivia questions (6 structure types * 3 questions) from the task set in the previous section based on the following formats of quiz. For example:

- Association: presenting several examples associated with something and asking what it is

(e.g., The ruins of Ilios, Cappadocia, and the Topkapi Palace are famous tourist spots in this country. Which country is it? --- [answer: Turkey])

- Common: explaining two or more items and asking the common name for them

(e.g., This refers to the highest seats in a theater, exhibition rooms in an art museum, and spectators in golf. What is this word? --- [answer: gallery])

- One of N-items: presenting N-1 items out of N items and asking for the remaining one

(e.g., The nutrients essential to the human body, known as the “three major nutrients,” are fats, proteins, and what? --- [answer: carbohydrates])

- Other names: presenting another name (such as a Japanese name, an etymology, or an official name) of something and asking what it is

(e.g., This is also known as a Petri dish. This is a shallow transparent container used for culturing bacteria. What is its German name? --- [answer: schale])

- Parallel: question with the phrase “It is ~, and/but...?”

(e.g., In English grammar, “I” is nominative case, “my” is possessive case, but “me” is what? --- [answer: objective case])

- Others: none of the above.

(e.g., Even when electric appliances are turned off, as long as they remain plugged in, a tiny amount of electricity is still consumed. What is this electricity called? --- [answer: standby power])

Our criteria for selecting the questions were as follows. First, we selected easy questions for which most trivia experts would know the correct answer if they hear the whole question text. Second, we included questions in which the critical cue that uniquely identifies the correct answer appears in various positions (i.e., the beginning, middle, or end of a question text). Third, we included various genres of questions such as science, history and vocabulary (and see also Supporting information 03 in S1 File).

Although quiz questions can be categorized into more types of structures [6], we focused on the above six structures because they are typical in quiz questions. Ideally, more questions might be prepared. However, considering participants’ workload in a behavioral experiment, we limited 18 questions (even with only 18 questions, the task took 30–60 minutes to complete). For the 18 questions we used, see Supporting information 03 in S1 File.

#### 2.2.3 Tasks and procedure.

**Fig 3:**
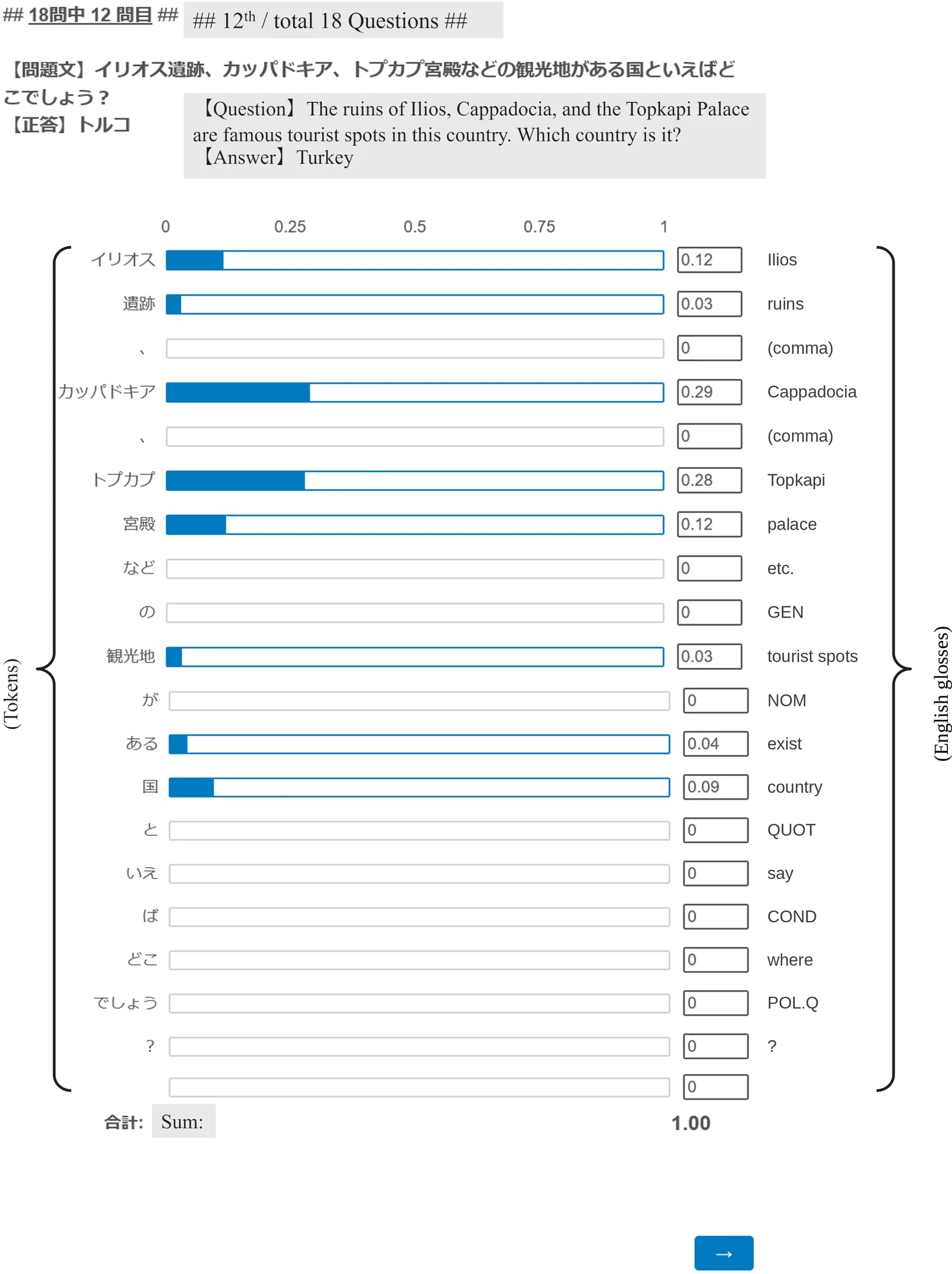
Screenshot of the behavioral experiment. Participants were asked to evaluate how important each token was for identifying the correct answer, using a visual analog scale. The gray-highlighted descriptions and the descriptions to the right of the bars are English translations (Japanese tokens and their English translations do not always correspond exactly one-to-one), and participants (all Japanese people) were not presented with them in the actual experiment.

The experiment was performed online via Qualtrics. The procedure of one trial was as follows. A question text and its answer were displayed on the top of a computer screen. Below them, each word/phrase of the question (tokenized by “Tokenization module” in the theoretical analyses) was also displayed. Next to each of the words, a slider bar for a visual analog scale was presented. Participants were asked to rate how useful each word was as a cue for identifying the correct answer using the slider, ranging from 0.00 to 1.00. The left end of the slider indicated 0.00 as “not useful at all”, while the right end indicated 1.00 as “very useful” (Fig 3). Participants were required to adjust the sum of the rating values to exactly 1.00 (the computer program was designed so that the sum of values could not exceed 1.00). Hereafter, we call this participants’ rating score “human’s evaluation score”. When the sum of the values reached 1.00, the “Next” button became active. When participants pressed the button, the next question was presented. There was no time limit for each question. The order of appearance of questions was randomized. After completing all questions, participants provided demographic data such as their age, gender, and quiz experiences (the number of years spent in competitive quizzes).

## 3. Results

### 3.1 Overall performance

**Fig 4:**
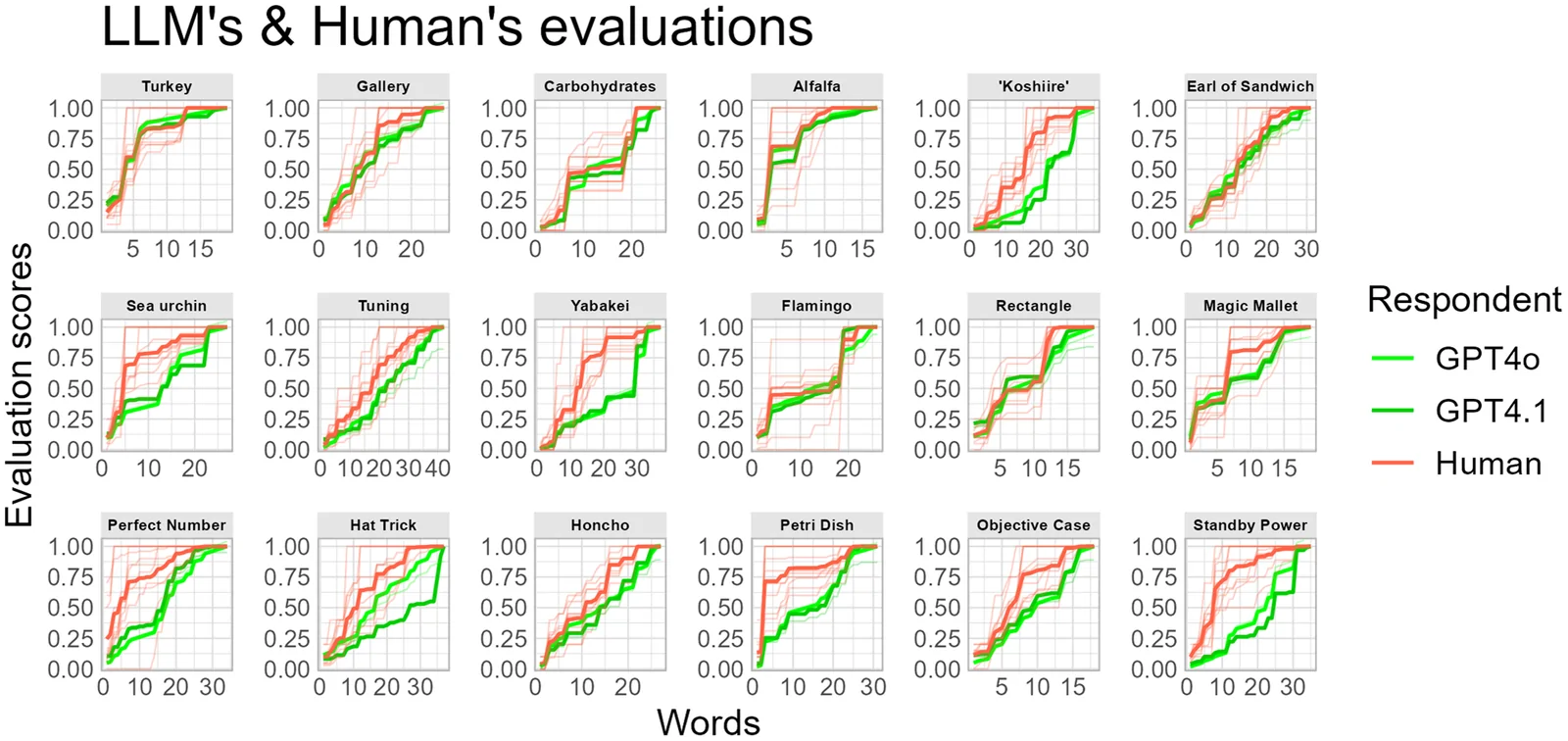
Cumulative evaluation scores. The x-axis shows the token number (i.e., when the word/phrase was presented in a question). The y-axis shows the cumulative evaluation scores by LLMs (two green lines) or by humans (red line). Each panel denotes each question, and the facet title in the gray bar represents the correct answer of the question.

To examine how trivia experts weighed information in quiz questions, we compared humans’ evaluation scores with those of the system. Specifically, we calculated their cumulative evaluation scores. Then using system’s evaluation scores as a comparative tool, we discussed words/phrases that humans (trivia experts) focus on in situations where information is obtained sequentially. As overall tendencies, many human participants appeared to accumulate scores faster than LLMs. This was because the red lines generally lay above the green lines at earlier token positions (Fig 4).

**Fig 5:**
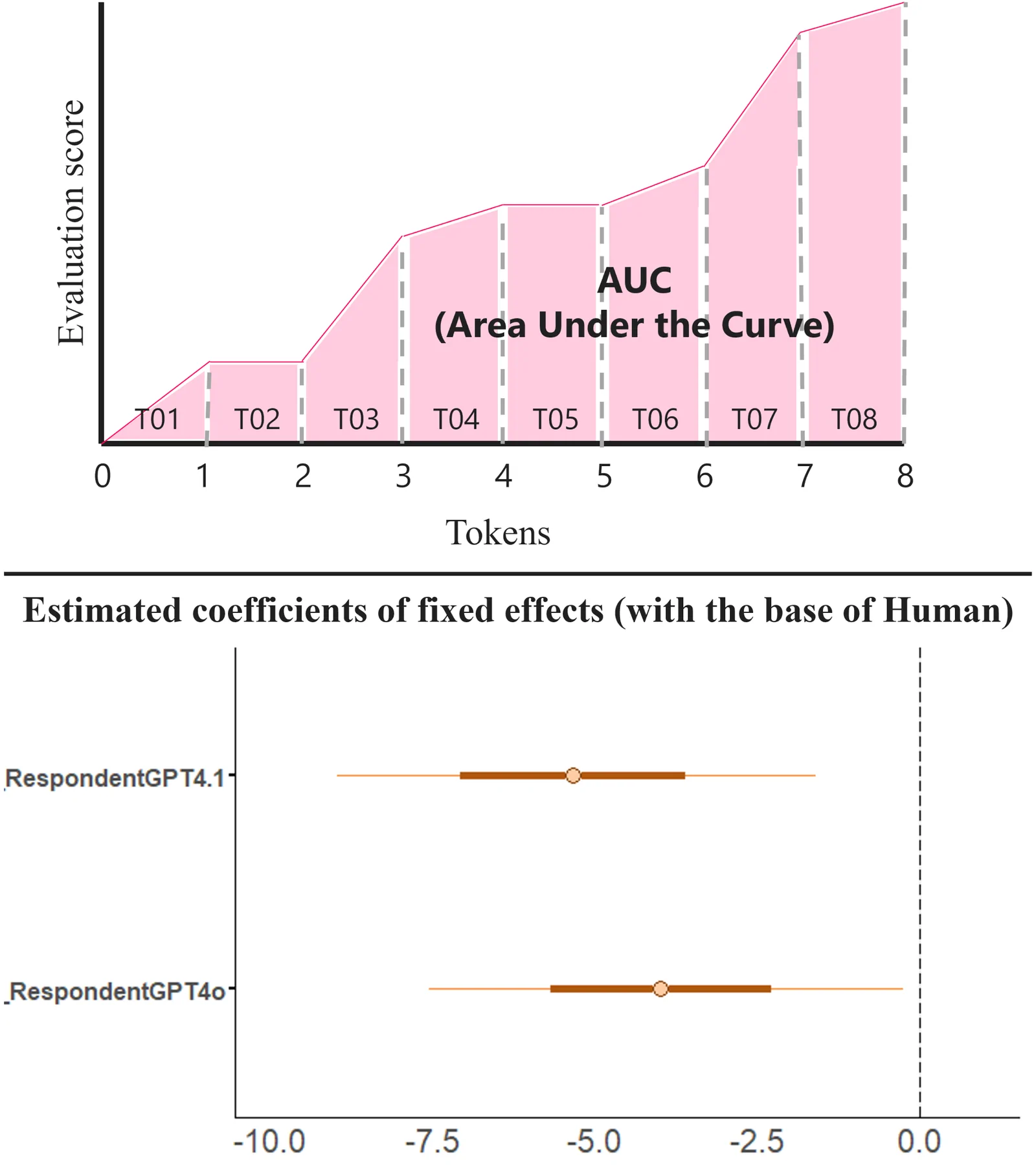
Schematics of AUC (area under the curve) and results of parameter estimations. Upper: AUC calculation in one question. We calculated trapezoids (example of a case where the number of tokens is eight) and regarded the sum of them (T01 ~ T08 in this example) as AUC in this question. Lower: 95% credible intervals (CIs) of coefficients of fixed effect (Human/GPT-4.1/GPT-4o) with the base of Human. The dots show the estimated coefficients. The bold and thin lines show 68% and 95% CIs, respectively.

To statistically confirm such tendencies, we calculated AUC (area under the curve) for each question as an index for the speed of score accumulation. Specifically, AUC was estimated by the sum of the areas of the trapezoids as follows [24]:


$$\begin{array}{c} \begin{array}{c} AUC=\frac{\text{1}}{\text{2}}\sum_{n=\text{2}}^{N}\left({score}_{n}+{score}_{n-\text{1}}\right)*\text{1}.\text{0}\text{0} \end{array} \end{array}$$

where *N* means the number of words (or tokens) and ${score}_{n}$ means the evaluation score at the *n*^th^ word in a certain question. The height of each trapezoid was fixed at 1.00 because the x-axis denotes token indices, which were in increments of 1.00 (for example, see Fig 5 upper panel). A larger AUC therefore indicates that the evaluation scores reached 1.00 faster.

We calculated the averaged AUC in humans (trivia experts), GPT-4o, and GPT-4.1 for each question (note that in some questions, LLMs’ cumulative evaluation scores did not exactly match 1.00 due to rounding off; thus, we normalized the scores for each question so that the maximum value is exactly 1.00). Then, we tested differences in AUC between them by a mixed model, wherein the dependent variable was AUC, the fixed effect was respondents (humans, GPT-4o, and GPT-4.1), and the random effects were questions and respondents. This analysis was conducted by brm() function in R package “brms” [25]. Parameters were estimated with 4,000 iterations, 2,000 warmup, and three chains. We found that with the base of humans, the estimated coefficients of the fixed effect were −3.98 (95%CI = [−7.56, −0.26]) for GPT-4o and −5.34 (95%CI = [−8.97, −1.61]) for GPT-4.1 (Fig 5 lower; for the details of estimation results, see Supporting information 04 in S1 File). The coefficient values were negative, and their 95% credible intervals did not overlap 0.00. Therefore, AUC in trivia experts was larger than that in these two GPTs. This indicates that experts’ cumulative evaluation scores exceeded those of the system at an earlier point.

### 3.2 Specific performance for each quiz structure

**Fig 6:**
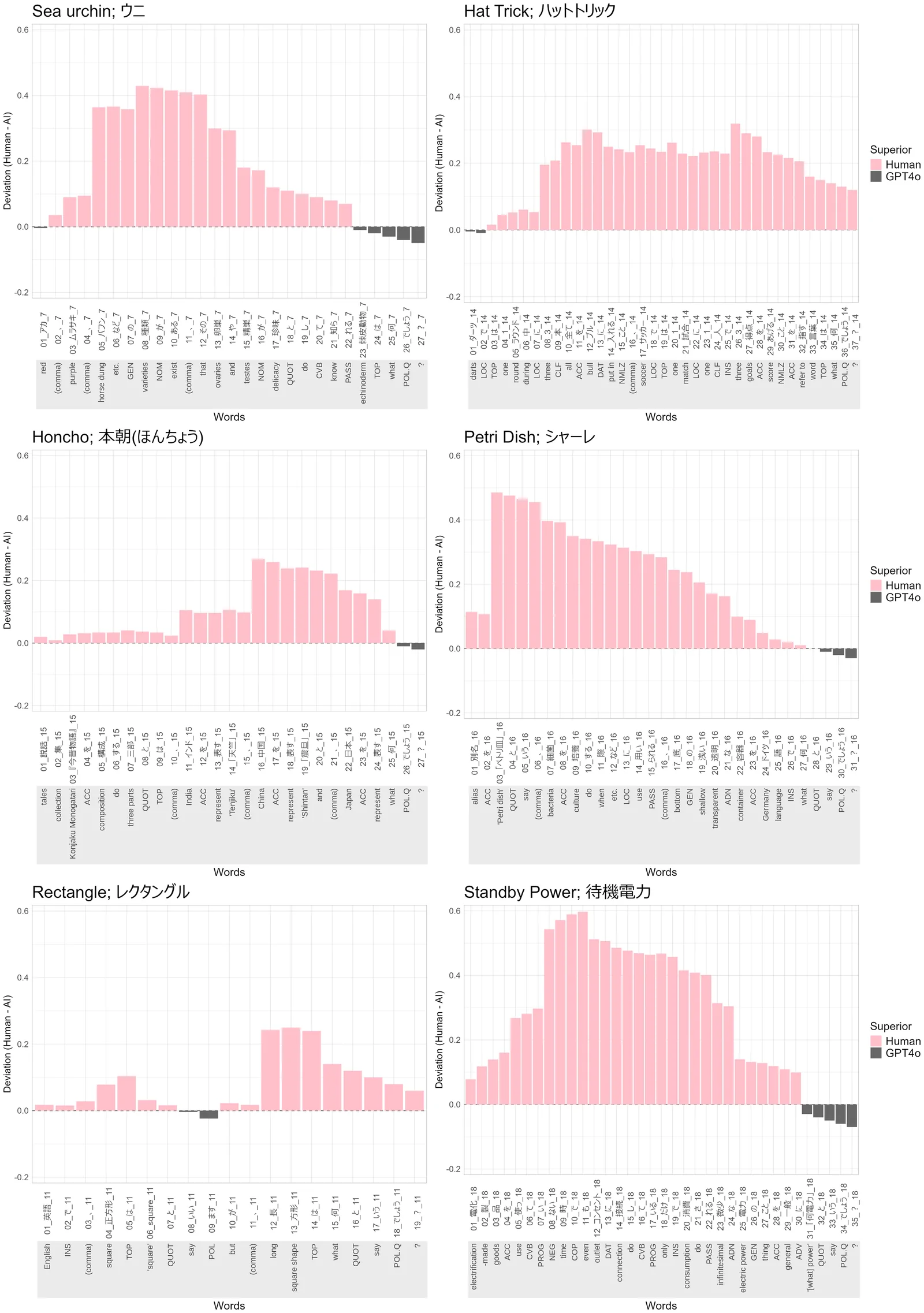
Examples of results of deviations between human’s (mean) and LLM’s (GPT-4o) evaluations. The x-axis shows tokens in a question (denoting “*token number*_token_*question id*”). The y-axis shows the deviations. The positive values (pink) denote that the mean of humans’ scores was larger than LLM’s score, while the negative values (black) denote that LLM’s score was larger than humans’ score. The gray-highlighted descriptions on the x-axis are the English translations of the Japanese tokens (Japanese tokens and their English translations do not always correspond exactly one-to-one).

In trivia experts’ evaluations, what tendencies were observed for each quiz structure? To examine characteristics of their evaluation for each word/phrase in more detail, we focus on each quiz structure as a set of case-based analyses. Here, we introduce an index “deviation,” which denotes the gap between trivia experts’ and system’s evaluation scores (i.e., gaps between red and green lines in Fig 4). Note that in the subsequent Fig 6, we visualize only some remarkable results between trivia experts and GPT-4o (similar tendencies were observed between GPT-4o and 4.1). For the deviations in all questions, see Supporting information 05 in S1 File.

**- Structure “association”**

Both trivia experts and LLMs could infer the correct answer based on a few examples associated with it. LLMs tended to regard words as more important if the presented words had strong associations with the answer (e.g., inclusion relationships like “The ruins of Ilios are in Turkey”). Importantly, however, experts could sometimes make faster judgments even based on words with weaker associations.

For example, in the question of “sea urchin (Fig 6, top left),” the words “アカ (‘red’ in English)” and “ムラサキ (‘purple’ in English)” generally indicate color names and have weak associations with “ウニ (‘sea urchin’ in English)”. LLMs were unlikely to evaluate such words as important, while experts could evaluate them as important cues that refer to some examples (or types), such as “アカウニ (red sea urchin)” and “ムラサキウニ (purple sea urchin)”. And when a more characteristic sea urchin type (“バフン”) was presented, experts appeared to become strongly confident that they were being asked about “sea urchin” (“バフンウニ” is often called ‘Japanese green sea urchin’ or ‘bafun sea urchin’ in English). In the question of “perfect number”, subjective evaluations also followed a curve similar to the question of “sea urchin” (see Fig 4, the middle and bottom panels in the left column), which suggests that experts made judgments similarly to infer “sea urchin.”

**- Structure “common”**

Trivia experts’ and LLMs’ evaluation scores tended to increase in a similar way. They seemed to gradually collect cues from the beginning to the end. Experts would have to seek the answer while considering what element is common to the listed items (e.g., the highest seats, exhibition room, and spectators). The similar tendency can be observed in the questions of “Gallery” and “Tuning”.

However, as in the question of “Hat trick” (Fig 6, top right), if words/phrases that can identify the answer appear early in a question, experts may be able to find the correct answer faster than LLMs. In quiz questions, more familiar and clear items (i.e., words/phrases strongly associated with the correct answer; e.g., soccer — hat trick) are generally often placed later in a question text. LLMs’ scores are therefore likely to increase as a question text progresses. In contrast, even if unfamiliar items (words/phrases are not always strongly associated with the answer; e.g., darts — hat trick) are placed in an earlier stage, experts can often utilize them as important cues if these words/phrases are sufficient to identify the correct answer.

**- Structure “one of N-items”**

Compared to LLMs, experts might focus more sensitively on a word/phrase at which the correct answer is uniquely identified. When a question concerns a category that contains N items, the correct answer is identified at the moment the N-1th item (or its first character) is presented in a question. For example, for considering “three major nutrients,” hearing up to “fats, p…” (“p” as in “protein”) is enough to identify the remaining item “carbohydrates” as the answer.

Similarly, in the question of “Yabakei”, the phrase “‘Onuma’ and ‘Miho no Matsubara’ and this” allows one to understand that the remaining “Yabakei” is asked. Thus, this phrase should be crucial in this question. In the question of “Honcho” (Fig 6, middle left), each item is presented not as a single word but as a phrase paired with multiple words, such as “India” with “Tenjiku.” Consequently, hearing the first character of the N-1th phrase, “中国 (‘China’ in English)”, allows one to understand that the question is asking “Japan is what?”. Therefore, the word “中国” should be highly crucial. Trivia experts’ evaluation scores were substantially higher than those of the LLMs at such words/phrases.

**- Structure “other names”**

Another name of something (e.g., also known as…; Japanese terminology; etymology) can directly indicate the answer. Thus, such a name should itself become a crucial cue for identifying the answer. Indeed, both trivia experts and LLMs tended to assign higher values to such words. In the questions of “Alfalfa” and “Flamingo”, they evaluated a term (e.g., “murasaki umagoyashi” for alfalfa) or an etymological source (e.g., “flame” for flamingo) as important. In these questions, experts and LLMs showed a similar curve (a sharp jump) at the beginning of the question (see Fig 4, fourth columns).

On the other hand, sometimes LLMs still seemed weaker than experts in associating such other name(s) with the proper names. In the question of “Schale” (Fig 6, middle right), the word “Petri dish” is another name for a schale and can serve as a highly critical cue. In fact, experts evaluated “Petri dish” as highly important. In contrast, LLMs did not evaluate “Petri dish” as highly important and gradually considered the answer from subsequent words/phrases such as “shallow container” and “culturing bacteria”. LLMs should have much more knowledge/information than humans and should *know* both Petri dish and schale. Nevertheless, it seems difficult for LLMs to judge that the petri dish is a critical cue for leading to schale. As also observed in “one of N-items” structure questions, experts may be able to capture the word/phrase at which the answer can be uniquely identified faster and more accurately than LLMs.

**- Structure “parallel”**

The system’s evaluation scores generally showed a linear increase. It can be interpreted that LLMs gradually collect cues for the answer from the beginning to the end, as in the “common” and “others” structures. In other words, LLMs seem not to effectively utilize the parallel structure.

Meanwhile, trivia experts assigned high values to the first word/phrase in the latter half of a sentence (i.e., right after a conjunction “but”; “...ですが、” or “...ますが、” in Japanese) or to a word/phrase even in the first half (i.e., before a conjunction “but”). For example, experts evaluated “my”, which was placed before a conjunction “but”, as a highly useful cue. This suggests that humans inferred at this point that the next item following “I, my, …” would be asked. To put it abstractly, humans can readily detect a structure such as “A is A’, B is B’, but C is what?”, and can predict that “the word C’ will ultimately be asked on hearing the word B”, as in the “one of N-items” structure. Also in the other two questions, “Koshiire” and “Rectangle”, subjective evaluations tended to increase even in earlier stages of the sentences. Especially in the “Rectangle” question (Fig 6, bottom left), hearing the word “長 (pronounced as ‘Chou’)” makes it clear that “正方形 (‘square’ in English; pronounced as ‘Seihoukei’)” and “長方形 (‘rectangle’ in English; pronounced as ‘Chouhoukei’)” will be contrasted. Thus, trivia experts may have regarded the initial word “長” as a highly important cue to identify the answer, utilizing the “parallel” question structure.

**- Structure “others”**

Unlike “one of N-items” or “other names” structures, it is difficult to clearly find words/phrases that can uniquely identify the correct answer. In such questions, no particular tendencies could be identified in trivia experts (compared with LLMs) at the word/phrase level.

However, as with many other quiz questions, experts’ evaluation scores appeared to reach 1.00 faster than the system’s scores. Focusing on individual tokens, experts placed importance not only on specific nouns but also on subtle words such as auxiliary verbs and adjectives. For example, in the question on “standby power” (Fig 6, bottom right), human subjective evaluation score increased markedly at a negation auxiliary verb “(使われて)いない” in Japanese (corresponding to ‘are turned off’ in English). Similarly, in the questions of “magic mallet” and “Sandwich”, participants rated “振れば (‘if one shakes’ in English)” and “...を挟んだ (‘are placed between’ in English)” as highly important, respectively.

These examples imply that trivia experts may be able to make faster and more accurate inferences by using any token of a question text as important cue(s), even when the individual tokens do not appear to be direct or crucial cues at first glance.

## 4. Interpretation of the quiz-structure results

The results of overall performance imply that humans often focus on various words/phrases that LLMs may overlook. The results for specific performance suggest that LLMs generally evaluate tokens that were strongly related to the correct answer as more important, regardless of quiz structures. Even if a token can uniquely identify the answer, LLMs sometimes judge it as less important if it seems to have weak associations with the answer.

Taken together, our case-based analyses suggest that trivia experts may (i) be able to focus more sensitively on words/phrases which can uniquely identify the answer; (ii) make fast and accurate inductive inferences about which generic concept is being asked from only a few types or examples; and (iii) often try to identify the answer from various words/phrases in a sentence even if they do not always seem to have a strong association with the answer. These tendencies may be one of the characteristics of human intelligence, which has much less knowledge than LLMs, in sequential information processing like buzzer quizzes.

## 5. General discussion

Compared to AI, humans should have many cognitive constraints such as limits on computational ability, memory capacity, and knowledge. However, humans can sometimes make judgments fast and accurately, even when information is limited or insufficient. In particular, human trivia experts can often respond to trivia questions from only a few words, which should be highly difficult for AI. Humans and AI would therefore be expected to differ in their evaluation of which words/phrases in a question text are important and to what extent they contribute to identifying the correct answer. In other words, humans might be more sensitive to important words/phrases than AI and might use a wider variety of words/phrases as cues. To address such issues, this study compared humans’ performance with LLMs’ performance in solving trivia quiz questions, through an LLM-as-a-judge approach. We constructed a quiz question processing system that tokenizes question texts based on morphological analysis and then numerically evaluates the importance of each token using two widely used LLMs, GPT-4o and GPT-4.1. Then, we asked human trivia experts to evaluate the importance of each word in questions through a behavioral experiment, just as the LLMs had done. By comparing LLMs’ and humans’ evaluations, we found that both humans and LLMs could sequentially and gradually accumulate evidence for the correct answer, but humans tended to accumulate it faster than LLMs. LLMs could generally regard tokens that were strongly associated with the answer as important, regardless of quiz structures. In contrast, humans could use various words/phrases as cues for predicting the subsequent words/phrases and the correct answer, even if they seemingly did not have strong associations with the correct answer. In other words, they could make inferences of the correct answer inductively even from only a few items (i.e., finding a common concept indicated by the already presented items).

### 5.1 Limitations

There are several limitations of this study. The first limitation is the small data. As in cases of expert studies such as those of professional dancers or magicians (e.g.[26,27]), the population of trivia experts is very limited, and it is difficult for them to participate in an experiment (Especially in quizzes, as most quiz players have their primary job outside of quizzes, it is sometimes difficult for them to devote their time to academic experiments). Due to the small sample size and the small number of questions in the behavioral experiment, the current results are only preliminary, and we should not overstate them. We investigated only trivia experts’ cognition, which cannot always be generalized to all humans. Therefore, we should capture our findings as results of case-based analyses. To strengthen robustness, further examinations are needed by increasing the sample size and the number of questions in the future.

The second limitation is the system validation. In the theoretical study, two quiz players briefly scanned our system’s outputs. This inspection may be acceptable within an exploratory framework, but more rigorous, objective and quantitative validation is needed to use LLM as a more reliable tool (see also [28]), such as scanning by more trivia experts and testing other models (see also below). Brief and qualitative checks in this study may affect the validity and applicability of our systems, and the generalizability of our findings. Therefore, further verification is required in the future. Relatedly, we should emphasize clearly that within the LLM-as-a-judge framework, this study used LLMs just as comparative analytical tools rather than as normative or ground-truth judges. We explored the characteristics of trivia experts’ cognition in quiz questions through LLMs, and LLMs’ validity as evaluators remains an open methodological question. That being said, we expect that this study will be the first step to highlight human intelligence to correctly and accurately respond to a question from limited information.

The third limitation is about experimental paradigms. One may argue that although this study targeted “fast and accurate judgments,” the absence of time pressure and the use of slider-based scoring in the experiment might induce participants’ slow and analytical processes. Thus, it is unclear whether the experiment captured faster intuitive decisions or slower deliberative evaluations. This mismatch between ecological validity and experimental design cannot be fully re-solved within the present study. That said, to some extent, this design may still reflect trivia experts’ thinking processes in actual competitive quiz situations. In fact, according to actual trivia experts (e.g.[6,10]), even in competitive situations where real-time and instantaneous judgments are required, players narrow down candidate answers to a single correct answer on the basis of information (word/phrase) that is gradually revealed. They also reported that the process from obtaining information to considering the answer sometimes feels extremely slow, almost like slow motion. Therefore, we believe that although it did not replicate competitive quiz situations, our behavioral experiment in which participants evaluated the importance of each individual word/phrase was reasonably suitable for experimentally investigating their thinking processes.

The fourth limitation is the generalizability across LLM architectures. To confirm the robustness of LLM’s evaluations, we manipulated some factors such as the temperature parameter and prompts (see the Methods section). However, our analysis was still limited to a single model family (GPT). It is more desirable to test at least one model from a different architecture such as Claude, Gemini, or an open-source model such as Llama. By using these models, we will be able to strengthen the robustness of our results.

The fifth limitation, partially related to the second limitation, is another methodological issue. In our experiments, participants were required to evaluate predefined, tokenized word/phrase units. In contrast, it is considered that in actual buzzer quizzes, humans are unlikely to process questions strictly on a word-by-word basis; instead, they may rely on more holistic and contextual interpretations. Thus, our experimental design might impose an artificial constraint on human cognitive processes and reflect a form of “human evaluation adapted to an LLM-oriented format” rather than natural human cognition. Experimental design in this area remains open to debate. However, human trivia experts often study quiz questions considering which word will be critical to identifying the correct answer faster, focusing on each word/phrase level even in actual buzzer quizzes [6]. Therefore, we believe that actual trivia experts’ processes could be more or less reflected in our experiment.

### 5.2 Practical implications

We now turn to possible future directions. This study investigated human intelligence compared to artificial intelligence, in terms of what words/phrases in quiz questions trivia experts selectively focus on when considering the correct answer from limited information. We believe that our findings can provide practical implications for improving real-world AI systems, such as AI for responding to customers’ questions. In call centers and customer support centers, answering customer inquiries has increasingly been automated using AI. However, customer questions are often incomplete sentences and are provided only with fragmented information. Findings of studies on human cognitive processes, like this study, will be useful for designing AI that can respond quickly and accurately to such questions [5] (see also [29]). If there are tokens (words/phrases) that many trivia experts evaluate as highly important but that LLMs do not, AI designers can make AI responses closer to human responses by re-designing prompts or parameters so that LLMs can evaluate such tokens as important. For example, AI designers could strengthen LLMs’ inductive reasoning so that it identifies commonalities when multiple items (sometimes, not a complete phrase but only fragmented keywords) are provided from a customer. Furthermore, if there are word pairs that are weakly associated in a language corpus but strongly associated by humans, AI designers can train LLMs to largely weight their association/similarity.

Note that one may argue that the “parallel” structure proposed in this study seems unique to quizzes (especially Japanese buzzer quizzes). However, even in the real world, people sometimes ask a question like the parallel structure, such as “A is A’ but B is what, again...?” (e.g., your last name was Smith, but what was your first name again?). The parallel structure can therefore be regarded as reflecting some real-world questions.

## 6. Conclusions

This study is the first attempt to reveal what words trivia experts selectively focus on through behavioral experiments. Our findings may provide scaffolding for developing more advanced AI systems and understanding AIs’ internal information processing. Achieving this will require future studies to investigate not only human intelligence but also artificial intelligence.

## Supporting information

S1 File Supporting Information 01 (DOCX)
