# Supplementary material for: Attempts to investigate what words trivia experts focus on in quiz questions: Human-AI comparison through “LLM-as-a-judge” approach
Source: PLoS One. 2026 Sep 22;21(9):e0358332. doi: 10.1371/journal.pone.0358332 (PMC13596818; doi:10.1371/journal.pone.0358332)
Supplement: Supplementary file 1 [file pone.0358332.s001.docx]

**Supporting Information**

**Attempts to investigate what words trivia experts focus on in quiz questions:**

**Human-AI comparison through “LLM-as-a-judge” approach**

\## All data, code, and materials used in our experiments are available at

- GitHub link (for system pipeline):

https://github.com/bravebird0914/llm-quiz-attribution

- OSF link (for appendix and behavioral data):

https://osf.io/hvfsn/overview?view_only=fb2037eaaaf04cbea271a207dbc84f7a

Note: The used language in database was all Japanese. It is highly difficult to translate all Japanese 2,480 questions into English, and it is impossible to translate Japanese into English word-by-word level completely. Therefore, parts of materials in this document include Japanese text. In this document, there is no identifying data such as name, initial, birthday, IP address, and so on.

# **Supporting Information 01**

\## Details of the quiz question processing system

**1. System Overview**

We implemented a “quiz question processing system” that uses Large Language Models (LLMs) to quantify the extent to which each token in a quiz question contributes to deriving the correct answer. The system first separates a question text into several words/phrases (i.e., tokenization) based on morphological analysis; and then evaluates the importance of each token. The later module used GPT-4o and GPT-4.1, with the same prompt design and hyperparameters to ensure comparability of outputs. The outputs are weights of importance for each token evaluated by LLMs.

**2. Data Preprocessing**

2.1 Input Data Structure:

Each sample is provided as a JSON object with the following keys.

{

"data_id": 2201,

"question": "イリオス遺跡、カッパドキア、トプカプ宮殿などの観光地がある国といえばどこでしょう？",

"answer": "トルコ",

"tokens": "|イリオス|遺跡|、|カッパドキア|、|トプカプ|宮殿|など|の|観光地|が|ある|国|と|いえ|ば|どこ|でしょう|？|",

"token_count": 19

}

2.2 Tokenization

To handle ruby annotations, diverse bracket structures, and long specialized terms characteristic in Japanese quiz questions, this study employs a multi-stage processing architecture based on MeCab morphological analysis. The pipeline consists of the following 4 phases:

1. Preprocessing Phase: Ruby removal and minor normalization

2. Basic Tokenization Phase: Initial segmentation using MeCab (extracting surface forms and part-of-speech tags)

3. Semantic Integration Phase: Recognition of parenthetical structures and recursive processing

4. Syntax Combination Phase: Adaptive combination based on part-of-speech tags and character types

The final token sequence is serialized into a pipe (|)-delimited string at output, ensuring token_count matches the token count (e.g., ‘|’ + ‘|’.join(tokens) + ‘|’). This format aligns with the input/output specifications in §2.1.

2.2.1 Preprocessing Phase: Ruby Removal and Normalization

Purpose: Remove ruby (reading guides) to enhance stability for subsequent morphological analysis and integration rules. (for Python code for this process, see “tokenize_system.py” in OSF)

○ Target Patterns

・Ruby within parentheses: Example: “宮殿（きゅうでん）” retains “宮殿” and removes the parenthetical reading.

・Ruby with pipes: Example: “|吉原|重俊(よしはら・しげとし)|” is simplified to “吉原重俊” (preserving the headword + main text).

○Ruby Determination Criteria (Heuristics)

・Number of characters within parentheses: Within 15 characters.

・Character type constraints: Only hiragana, katakana, prolonged sound mark “ー”, and bullet “・”.

・Parent character constraints: The immediately preceding parent character group must consist of kanji, katakana, alphanumeric characters, or certain symbols.

○Character Type Identification

・Identify hiragana (U+3041–U+3096), katakana (U+30A1–U+30F6), kanji (U+4E00–U+9FAF), and alphanumeric characters (A–Z, a–z) based on Unicode ranges. Internally store word type information for reference in subsequent combining rules.

○Design Intent

・Ruby removal prevents strings containing ruby from inducing redundant segmentation or incorrect merging, which enables us to smoothly apply subsequent MeCab analysis and integration rules.

2.2.2 Basic Tokenization Phase: MeCab Morphological Analysis

Purpose: Perform initial segmentation to obtain pairs of surface forms (actual strings) and part-of-speech tags.

・Processing: Scan the entire text with MeCab, excluding BOS/EOS nodes. Extract surface forms and part-of-speech tags (noun, verb, adjective, particle, symbol, etc.) from each node to obtain a sequence.

・Output Format (Internal Representation): [(Surface Form, Part-of-Speech Tag), …].

・Notes: At this stage, segmentation is limited to lexical level; excessive segmentation of proper nouns and compound nouns is suppressed in the subsequent integration phase.

2.2.3 Semantic Integration Phase: Recognition and Recursive Processing of Bracket Structures

Purpose: Normalize diverse bracket expressions in Japanese as units, and recursively tokenize their internals as needed.

・Corresponding Pairs: e.g., 「…」, 『…』, （…）, (…), […], ｛…｝, {…}.

○Recursive Tokenization Threshold (applied to angle brackets):

・If content within brackets is 7 characters or more: Analyze internal structure (perform recursive tokenization).

・If 6 characters or fewer: Retain as a single unit (proper noun or phrase).

// Note that the threshold of 7 characters is an empirical standard based on the distribution of short and long proper expressions in Japanese quizzes. It avoids disruptive splitting of short proper nouns while enabling internal analysis of long quotations or explanatory text.

○ Policy by bracket type:

・Angle brackets (「」「『』): Only long texts undergo internal recursive tokenization based on the above threshold.

・Round brackets (() / ()): Merge with the preceding token as a single unit (e.g., “漢字(かんじ)” is unified into one word).

・ Square brackets and curly brackets ([] / {} / ｛｝): Generally retained as single units.

○ Handling Unsupported or Single-Side Missing Brackets

・When brackets are unbalanced, treat the symbol as a standalone token to prevent semantic breakdown spreading to the subsequent text.

2.2.4 Syntax Combination Phase: Adaptive Integration Based on Part-of-Speech and Character Type

Purpose: Recombine excessively split words using rules based on part-of-speech sequences and character type sequences to restore compound words and technical terms common in quiz questions.

○ Combination Rules (in order of priority)

1. Consecutive part-of-speech match + character type match: Combine when consecutive elements are nouns, adjectives, or verbs and share the same character type (hiragana/katakana/kanji).

2. Consecutive Nouns (Phrase Constraint): Combine consecutive nouns where one is a phrase of two characters or fewer (e.g., abbreviations or linked conjunctive nouns).

3. Affix Relationships: Combine combinations containing affixes, such as prefix + noun or noun + suffix.

○ Special case for parentheses: Tokens inside parentheses are not combined. This preserves the autonomy of parentheses and prevents excessive fusion.

○ Tie-breaking rules: When multiple rules are simultaneously applicable, they are resolved by priority. If still tied, the order of occurrence (ascending) determines the result. This guarantees deterministic outcomes.

2.2.5 Post-processing Phase: Final Integration of Parentheses and Notation Formatting

・Parentheses Combination: When parentheses contain readings or annotations, they merge with the preceding token into a single token (e.g., “漢字(かんじ)” → “漢字(かんじ)”).

・Notation Formatting: Perform minimal formatting, such as removing unnecessary whitespace and normalizing consecutive symbols, without compromising output consistency.

・Output Format: Store the final token sequence as a |-delimited string in the tokens key, with token_count providing the token count (consistent with §2.1).

2.2.6 Internal Tokenization Subsystem (For Bracket Content Only)

Purpose: To balance local readability and accuracy for lengthy content within parentheses (meeting the threshold in §2.2.3).

Policy: Perform internal parsing using MeCab, retaining only punctuation marks (“,” “.”) as exceptions; remove all other symbols as a rule.

・Part-of-speech information: At the final stage of internal processing, retain only surface forms and discard part-of-speech tags (to avoid conflicts with outer integration rules).

・Recursion control: Limit recursion to a maximum of two levels to prevent excessive computational resource consumption and infinite loops, even for deeply nested citations or footnotes.

// Internal tokenization applies only to curly braces (“…”, “‘…’”), with a recursion depth limit of 2. Here, the recursion depth is counted from the outermost level by the number of nested brackets, k = 0 (directly under the main text), k = 1 (inside “...”), and k = 2 (inside “...『...』...”). For cases where k > 2 (e.g., “...『...「...」...』...”), recursion is terminated, and the bracket pair and its inner string are retained as a single unit. Round brackets are not subject to recursion and are combined with the preceding word in the post-processing phase.

2.2.7 Character Type Determination and Fallback

・Unicode range: Hiragana U+3041–U+3096, Katakana U+30A1–U+30F6, Kanji U+4E00–U+9FAF, English letters A–Z / a–z.

・Treatment of mixed words and unknown words: For words with unstable morphological analysis, if they contain kanji, katakana, alphabetic characters, or numbers, or if they are single characters, they are adopted as tokens. This ensures stable segmentation even for foreign words, specialized abbreviations, and proper nouns mixed with symbols.

2.2.8 Complexity and Performance Characteristics

・Time Complexity: Generally *O*(*n* × *m*) (*n*: number of characters, *m*: average search width of combination candidates).

・Space Complexity: *O*(*n*) (proportional to input length).

・Recursion Depth: Maximum two levels (for subsystems within parentheses).

・Operational characteristics: Exhibits near-linear time behavior for average quiz question lengths, maintaining stable throughput even during multi-item batch processing.

2.2.9 Quality Verification Items (Tokenization)

・Token boundary consistency: For the same input, deterministic token sequences are reproducible based on rule application order and tie-breaking rules.

・Bracket Pairing Completeness: Recognition rate of matching pairs and confirmation of fail-safe behavior when unmatched.

・Part-of-Speech Information Retention Rate: Assignment and maintenance of part-of-speech tags in the internal representation (final tokens are surface form sequences).

・Linearity of Processing Time: Confirmed to increase proportionally with input length in actual measurements.

・Output Consistency: token_count == len(tokens) and compliance with the consistency verification in §7.2.

2.2.10 Design Notes

This tokenization does not directly adopt the results of the morphological analysis. Instead, it selectively recombines elements from both semantic (parentheses) and syntax (part-of-speech, character type) perspectives. This yields Japanese token sequences robust to compound words, specialized terms, and ruby annotations.

Output is consistently pipe-delimited (‘|…|’), enabling seamless connection to subsequent weighting (Section 3 and after) and CSV output (Section 9) without modification.

**3. Prompt Design**

3.1 Structured Prompt

Provide the following structured instructions for the LLM. Placeholders {...} are replaced with input.

・Task: Evaluate the importance of each token in a quiz question

・Question text: {question}

・Correct answer: {answer}

・Token list: {token_list}

・Instructions:

1. Assign values close to 0 to tokens providing no clues,

2. Assign values close to 1 to highly useful tokens,

3. Adjust the total sum of all token weights to 1.00,

4. Output to two decimal places,

5. Return in JSON format.

Python Template Function Example:

def create_prompt(question: str, answer: str, token_list: list[str]) -> str:

return (

"【タスク】クイズ問題のトークンごとの重要度評価\n"

f"【問題文】{question}\n"

f"【正答】{answer}\n"

f"【トークン一覧】{token_list}\n"

"【指示】\n"

"- 正答を導くうえで全く手がかりにならないもの: 0に近い値\n"

"- 正答を導くうえで非常に有用な手がかりとなるもの: 1に近い値\n"

"- 各トークンの重要度の合計は必ず1.0になるように調整\n"

"- 小数第2位まで出力\n"

"- 出力はJSON形式のみ\n"

)

3.2 System Messages

Set the role definition “You are a trivia expert. Accurately evaluate the importance of the given tokens.” (Japanese original text: “あなたはクイズ問題の専門家です。与えられたトークンの重要度を正確に評価してください。”) for the system role to promote consistency in evaluation criteria.

**4. Model Invocation and Configuration**

4.1 API Call

We used identical settings for both models. Set temperature = 0.1 to increase determinism and max_tokens = 2000 for output length.

response = client.chat.completions.create(

model = "gpt-4.1", # model name “gpt-4o” or “gpt-4.1”

messages=[

{"role": "system",

"content": "あなたはクイズ問題の専門家です。与えられたトークンの重要度を正確に評価してください。"},

{"role": "user", "content": create_prompt(question, answer, token_list)}

],

temperature=0.1,

max_tokens=2000

)

4.2 Controlling Model Comparisons

We fully standardized prompt strings, system messages, hyperparameters, and input/output post-processing (§5–§6): All researchers have to do is to switch the model name “gpt-4o” or “gpt-4.1”.

**5. Response Analysis (JSON Extraction)**

To handle LLM responses containing code fences (json ...), extract the JSON body using regular expressions.

import json, re

def extract_json(text: str) -> dict:

m = re.search(r"```(?:json)?\s*(\{.*\})\s*```", text, flags=re.DOTALL)

payload = m.group(1) if m else text

return json.loads(payload)

After extraction, obtain attention_weights (array of tokens and evaluation scores).

**6. Numerical Processing**

We conducted normalization, rounding, and sum adjustment for the evaluation scores. The purpose of them was to obtain an evaluation-score sequence where values are discretized to two decimal places, and the overall sum is exactly 1.00.

We input a sequence of evaluation scores corresponding to each token. And then,

1. Non-negative clipping: Replace any negative weight values with 0.

2. Normalization: If the sum of weights is 0, divide equally by the number of elements and set each element to the same value so the total sum is 1. If the sum is not 0, scale each element by dividing by the total sum so the overall sum is 1.

3. Two-digit Discretization (Preprocessing): To prepare for discretization to two decimal places, multiply each value by 100, retaining both the integer and fractional parts without rounding.

4. Converting integers (Maximum Residue Method):

○ First adopt the integer part (truncated) of each value as the base allocation.

○ For the shortfall until the sum reaches 100, sequentially add 1 to the values in descending order of their fractional parts (in case of ties, use a fixed tiebreaker rule like ascending index order to ensure determinism).

5. Restoration: Divide the integer values obtained in step 4 back by 100 to form a weight column accurate to the second decimal place.

・Verification Conditions:

● The sum of all elements is exactly 1.00.

● Each element falls within the range 0.00 to 1.00.

● By fixing the clipping and allocation rules, deterministic output is obtained for the same input.

Implementation Example:

def normalize_to_1_two_decimals(weights: list[float]) -> list[float]:

import math

w = [max(0.0, float(x)) for x in weights]

s = sum(w)

if s == 0:

w = [1.0 / len(w)] * len(w)

else:

w = [x / s for x in w]

scaled = [x * 100.0 for x in w]

floors = [math.floor(x) for x in scaled]

remainders = [x - f for x, f in zip(scaled, floors)]

total = sum(floors)

need = int(100 - total)

order = sorted(range(len(w)), key=lambda i: remainders[i], reverse=True)

for i in range(need):

floors[order[i]] += 1

return [f / 100.0 for f in floors]

**7. Data Structuring and Verification**

The final output for one sample contained the following keys.

{

"data_id": 2201,

"question": "...",

"answer": "トルコ",

"tokens": "|...|",

"token_count": 19,

"attention_weights": [

{"token": "イリオス", "weight": 0.22},

{"token": "遺跡", "weight": 0.05},

{"token": "、", "weight": 0.00},

{"token": "カッパドキア", "weight": 0.32},

{"token": "トプカプ", "weight": 0.18}

// (… continue for all tokens)

],

"total_weight": 1.00,

"model_name": "gpt-4.1",

"model_response": "< LLM original responses >"

}

We verified the consistency in terms of (i) sum(w_i) must be 1.00 (abs(sum-1.00) < 1e-9 to account for floating-point error); (ii) len(attention_weights) == len(token_list); (iii) token_count == len(token_list) (corrected if mismatched); and (iv) Each score fell within [0.00, 1.00].

**8. Error Handling and Retries**

We also prepared for JSON Parsing Failures, API limits, and temporary errors in the following ways:

try:

data = extract_json(response_text)

except json.JSONDecodeError:

logger.warning(f"data_id={data_id}: 応答がJSON形式ではない")

logger.debug(f"raw_response={response_text}")

return None

Note: Use exponential backoff (maximum retry count as K, base delay as delay).

Note: Insert a default 2.0-second interval between items during batch processing.

import time, random

def with_retry(fn, K=5, delay=2.0):

for i in range(K):

try:

return fn()

except Exception as e:

if i == K - 1:

raise

sleep = delay * (2 ** i) + random.uniform(0, 0.3)

time.sleep(sleep)

**9. Output Conversion (CSV)**

9.1 Conversion to Single-Line Format

Normalize each sample to a single line, storing the token column and weight column separated by |.

def convert_to_csv(record: dict) -> tuple[str, ...]:

tokens = "|" + "|".join([aw["token"] for aw in record["attention_weights"]]) + "|"

weights = "|" + "|".join([f'{aw["weight"]:.2f}' for aw in record["attention_weights"]]) + "|"

return (

str(record["data_id"]),

record["question"],

record["answer"],

tokens,

weights,

str(len(record["attention_weights"]))

)

Output Example

2201,"イリオス遺跡、カッパドキア...",トルコ,"|イリオス|遺跡|...|","|0.22|0.05|...|",19

9.2 Post-Conversion Verification

For any row in the CSV, verify that row[3].count(‘|’) - 1 == row[4].count(‘|’) - 1 == int(row[5]).

**10. Pipeline Pseudocode (Overall)**

Input: data_id, question, answer, tokens_str

1: token_list ← split tokens_str by '|', remove empties

2: assert or fix token_count ← len(token_list)

3: prompt ← create_prompt(question, answer, token_list)

4: response ← chat_completions(model ∈ {gpt-4o, gpt-4.1}, prompt, temperature=0.1)

5: json ← extract_json(response.text)

6: raw_weights ← [json["attention_weights"][i]["weight"] for i in tokens]

7: norm_weights ← normalize_to_1_two_decimals(raw_weights)	▷ §6.2

8: attention_weights ← zip(token_list, norm_weights)

9: total_weight ← sum(norm_weights) (= 1.00)

10: export JSON/CSV and run format verification

Output: structured record per sample

**11. Reproducibility and Recording**

・Model/Setting Fixation: Record model_name, temperature, max_tokens, and prompt texts.

・Randomness Reduction: Set temperature = 0.1 and maintain identical settings for retries.

・Audit Log: Record question index (data_id), timestamp, model name, total weight, and validation result (success/fail) per row.

**12. Implementation Notes (Japanese Tokens)**

Our method assumes explicit lexical tokens, including punctuation and particles. By preserving presentation order without relying on morphological analysis, it clarifies the correspondence between model decisions and output scores.

Through the above steps, we can stably and reproducibly provide token-level importance distributions for any quiz question. Output scores in one question are normalized, rounded and sum-corrected, and then satisfy the constraint for sum 1.00.

# **Supporting Information 02**

\## Robustness of system outputs

As reported in the main text, we ran the LLMs by providing a prompt of a role (as “a trivia expert”) with temperature = 0.1. However, to confirm our methodological robustness, we also manipulated prompts of a role and values of temperature parameter using GPT-4.1 (note that, unfortunately, the official support for GPT-4o had ended when we conducted this additional analysis, Feb 18-28 2026; so, we had to use only GPT-4.1).

Specifically, we excluded “You are a trivia expert” from the prompt and simply provided “Accurately evaluate the importance of the given tokens.” (No-prompt version). This allowed us to confirm that LLMs’ performance was driven by an inference ability by LLM itself rather than by a role prompt. We also tried to range values of temperature to a default, 0.8 (default-temperature version). This could represent more randomness of LLMs’ outputs. Here, we report four conditions in total: Providing prompt and 0.1 temperature (reported in the main text); providing prompt and default temperature; providing no-prompt and 0.1 temperature; and providing no-prompt and default temperature. For each condition, we ran ten times and calculated the averages of each token’s evaluation score in the ten runs (note that the reason for the number of runs, ten, was that approximately ten players compete in quizzes in one round in many actual buzzer-quiz competitions).

As shown in the following figure, LLMs’ performance did not differ much regardless of these manipulations. That is, we could confirm methodological robustness.

**Results of LLMs’ robustness check across four conditions**. The x-axis shows the token number (i.e., when the word/phrase was presented in a question). The y-axis shows the cumulative evaluation scores by LLMs. We conducted the following four conditions: providing prompt and 0.1 temperature (“**Prompt, Temp 0.1**”); providing prompt and default temperature (“**Prompt, Temp Default**”); providing no-prompt and 0.1 temperature (“**NoPrompt, Temp 0.1**”); and providing no-prompt and default temperature (“**NoPrompt, Temp Default**”). Each panel denotes each question, and the facet title in the gray bar represents the correct answer of the question.

\## Absolute deviations from “**Prompt_Temp0.1**” for each question

| Answer_En | **Prompt_TempDef** | **NoPrompt_Temp0.1** | **NoPrompt_TempDef** |
| --- | --- | --- | --- |
| Turkey | 0.009826 | 0.007447 | 0.013409 |
| Gallery | 0.008981 | 0.026797 | 0.011923 |
| Carbohydrates | 0.014286 | 0.042543 | 0.034455 |
| Alfalfa | 0.019073 | 0.006501 | 0.011276 |
| 'Koshiire' | 0.011490 | 0.015986 | 0.017451 |
| Earl of Sandwich | 0.008479 | 0.039389 | 0.040536 |
| Sea urchin | 0.015807 | 0.041101 | 0.036705 |
| Tuning | 0.006623 | 0.011933 | 0.011682 |
| Yabakei | 0.013387 | 0.025400 | 0.037948 |
| Flamingo | 0.017928 | 0.003146 | 0.020213 |
| Rectangle | 0.044320 | 0.036047 | 0.065112 |
| Magic Mallet | 0.015521 | 0.002625 | 0.022600 |
| Perfect Number | 0.015793 | 0.014481 | 0.037240 |
| Hat Trick | 0.024885 | 0.120589 | 0.096913 |
| Honcho | 0.046463 | 0.041295 | 0.062652 |
| Petri Dish | 0.010859 | 0.017313 | 0.009555 |
| Objective Case | 0.013710 | 0.013065 | 0.024954 |
| Standby Power | 0.029615 | 0.016205 | 0.007705 |

# **Supporting Information 03**

\## Eighteen questions we used in the behavioral experiment

Note: the six structures of each question (see the main text) cannot always be deterministic, and quiz questions are often subjectively categorized into the structures. In this study, two trivia experts (one was the first author) categorized 18 questions into the above six structures and agreed on their categorization.

\### Words in the square brackets denote the structure of the question. These structure names are not provided in the actual buzzer quiz. Japanese texts follow English texts.

1. **The ruins of Ilios, Cappadocia, and the Topkapi Palace are famous tourist spots in this country. Which country is it? [association**]

*Answer: Turkey*

2. **This is the highest seat in a theater, an exhibition room in an art museum, and a spectator in golf. What is this word? [common]**

*Answer: Gallery*

3. **The nutrients essential to the human body, known as the “three major nutrients,” are fats, proteins, and what? [one of N-items]**

*Answer: Carbohydrates*

4. **A Japanese terminology of this is “murasaki umagoyashi”. This is a perennial plant of the legume family. What is this plant? [other names]**

*Answer: Alfalfa (Medicago sativa)*

5. **In Japanese words, favoring a specific person is called “kata-ire”, but getting married is, by using the name of a vehicle, called “WHAT-ire”? [parallel]**

*Answer: Koshi-ire*

6. **During the American Revolutionary War, this Earl served as British Minister of the Navy. A food in which meat and vegetables are placed between slices of bread is named after him. Who is he? [others]**

*Answer: Earl of Sandwich*

7. **Aka, Murasaki, and Bafun are types of this. An ovary and testis of this are eaten as a delicacy. What is this echinoderm? [association]**

*Answer: Sea urchin*

8. **Improving the function of car parts, matching the frequency of a radio, and adjusting the pitch of a musical instrument all share the same English word. What is it? [common]**

*Answer: Tuning*

9. **A person who visited here, Rai San-yo, named this. “Onuma” and “Miho no Matsubara” and this site are called “The New Three Views of Japan”. This site is located in Nakatsu-shi, Oita prefecture. What is the name of this valley? [one of N-items]**

*Answer: Yabakei*

10. **The Latin word for “Flame” is the origin of its name. A Japanese terminology of this is “benizuru”. What is this bird? [other names]**

*Answer: Flamingo*

11. **In English, “Seihoukei” in Japanese is called a “square,” but “Chouhoukei” in Japanese is called what? [parallel]**

*Answer: Rectangle*

12. **In the fairy tale *Issunbōshi*, if one shakes this, any wish will come true. What is this tool? [others]**

*Answer: Uchide no Kozuchi (Magic mallet)*

13. **8128, 496, 28, and 6 are examples of this. The sum of their proper divisors equals the number itself. What are such numbers called? [association]**

*Answer: Perfect numbers*

14. **In darts, it means throwing all three darts into the bull in one round. In soccer, it means scoring three goals by one player in one match. What is this term? [common]**

*Answer: Hat trick*

15. **The anthology of classical tales “*Konjaku Monogatari*” is divided into three sections: India is represented as “Tenjiku”, China is represented as “Shintan”, and Japan is represented as what? [one of N-items]**

*Answer: Honchō*

16. **This is also known as a Petri dish. This is a shallow transparent container used for culturing bacteria. What is its German name? [other names]**

*Answer: Schale*

17. **In English grammar, “I” is nominative case, “my” is possessive case, but “me” is what? [parallel]**

*Answer: Objective case*

18. **Even when electric appliances are turned off, only if they remain plugged in, a tiny amount of electricity is consumed. What is this electricity called? [others]**

*Answer: Standby power*

\### Japanese original texts

1. **イリオス遺跡、カッパドキア、トプカプ宮殿などの観光地がある国といえばどこでしょう？**

トルコ

2. **劇場の天井桟敷、美術展の画廊、ゴルフ競技の観客を指す時に共通して用いられる英単語は何でしょう？**

ギャラリー

3. **人間の身体に必要な「三大栄養素」と呼ばれる3つの栄養素とは、脂質、たんぱく質と何でしょう？**

炭水化物(たんすいかぶつ)

4. **和名をムラサキウマゴヤシという、マメ科の多年性植物といえば何でしょう？**

アルファルファ

5. **特定の人をひいきすることは「肩入れ」といいますが、お嫁に行くことをある乗り物の名を使って｢何入れ」というでしょう？**

輿(こし)入れ

6. **アメリカ独立戦争時にはイギリス海軍大臣を務めた伯爵で、パンに肉や野菜を挟んだ食べ物にその名を残すのは誰でしょう？**

サンドウィッチ伯爵

7. **アカ、ムラサキ、バフンなどの種類がある、その卵巣や精巣が珍味として知られる棘皮動物(きょくひどうぶつ)は何でしょう？**

ウニ

8. **車の部品などを改良して機能を高めること、ラジオなどでは周波数を合わせること、音楽では楽器の音の高さを合わせることに共通する英語は何でしょう？**

チューニング

9. **この地を訪れた頼山陽が命名した、大沼、三保の松原とともに新日本三景の一つに数えられている大分県中津市にある渓谷は何でしょう？**

耶馬渓(やばけい)

10. **ラテン語で「炎」という意味の言葉に由来する名前を持つ、和名を「ベニヅル」という鳥は何でしょう？**

フラミンゴ

11. **英語で、正方形はsquareといいますが、長方形は何というでしょう？**

レクタングル

12. **御伽草子『一寸法師』に登場する、振れば何でも願いが叶う道具は何でしょう？**

打出の小槌

13. **8128、496、28、6などがこれにあたる、その数自身を除く約数の和がその数と同じになる数を何というでしょう？**

完全数(かんぜんすう)

14. **ダーツでは1ラウンド中に3本全てをブルに入れること、サッカーでは1試合に1人で3得点をあげることを指す言葉は何でしょう？**

ハットトリック

15. **説話集『今昔物語』を構成する三部とは、インドを表す「天竺」、中国を表す「震旦(しんたん)」と、日本を表す何でしょう？**

本朝(ほんちょう)

16. **別名を「ペトリ皿」という、細菌を培養する際などに用いられる、底の浅い透明な容器をドイツ語で何というでしょう？**

シャーレ

17. **英文法で、Iは主格、myは所有格ですが、meは何格でしょう？**

目的格

18. **電化製品を使っていない時でもコンセントに接続しているだけで消費される微少な電力のことを一般に「何電力」というでしょう？**

待機電力

Note: besides the criteria that we described in the main text, we selected these questions also based on the clustering analyses. It is considered that there are various patterns in quiz questions in terms of when the critical and important word(s) will appear. In some questions, an important word appears at the beginning of the question text (e.g., in the question of “schale”, the phrase “(also known as) Petri dish” can be critical and uniquely identify the answer “schale” even at the beginning of the text.); in other questions, such a word appears at the end of the text (e.g., in the question of “Sandwich”, the clear hint “meat and vegetables … between slices of bread” is placed later in the question); or, neither of them (e.g., in the question of “magic mallet”, the individual words will not be very clear hints and players will gradually figure out the answer by hearing more information.). Thus, before selecting the above 18 questions, we conducted cluster analyses on all the 2480 questions (exploratory clustering with Ward method) based on the cumulative system’s evaluation scores. We normalized the number of tokens as 49, which was the longest number of tokens, among all questions (cumulative evaluation scores were interpolated linearly). We found that using R packages “cluster” and “NbClust”, the questions were categorized into three clusters, which could roughly be interpreted as the three patterns described above, respectively. Then, we adopted 18 questions for the behavioral experiment such that exactly six questions were selected from each cluster (for cluster numbers of each question, see the columns “clusterIdxAI” in the data “expVasAw4041GptsNaOmit.csv”).

**Results of cluster analyses among 2480 questions based on cumulative system’s evaluation scores**. The x- and y-axes show the token index and evaluation scores, respectively. Each line denotes each question, and bold lines denote their averages. The 829, 678, and 973 questions were clustered into Cluster 1 (red), 2 (blue), and 3 (green), respectively.

# **Supporting Information 04**

\## The descriptive statistics of the behavioral experiment

\### the mean & SD of token index at the time the evaluation scores reached 1.00 in 18 questions

\### (indicating how fast humans/LLMs accumulated the evidence for the answer)

|  | Mean | SD |
| --- | --- | --- |
| Humans (average of 11 experts) | 18.7 | 8.18 |
| GPT-4o | 28.1 | 7.63 |
| GPT-4.1 | 26.2 | 7.05 |

\### the mean & SD of AUC in humans/LLMs

\### (larger AUC could be interpreted as faster increase of evaluation scores)

|  | Mean | SD |
| --- | --- | --- |
| Humans (average of 11 experts) | 18.8 | 6.04 |
| GPT-4o | 14.8 | 3.29 |
| GPT-4.1 | 13.4 | 2.65 |

\### consideration of random effects of mixed models (to analyze differences in AUC)

AIC(lmer(data = questionAUChuman4041,

formula = AUC ~ Respondent + (subIdx|qIdx)))

\# boundary (singular) fit: see help('isSingular') # [1] 1209.049

AIC(lmer(data = questionAUChuman4041,

formula = AUC ~ Respondent + (subIdx|qIdx) + (1|subIdx)))

\# boundary (singular) fit: see help('isSingular') # [1] 1188.729

AIC(lmer(data = questionAUChuman4041,

formula = AUC ~ Respondent + (1|qIdx) + (1|subIdx)))

\# [1] 1218.408

AIC(lmer(data = questionAUChuman4041,

formula = AUC ~ Respondent + (subIdx|1) + (1|qIdx)))

\# [1] 1240.763

\### therefore, the third model "**AUC ~ Respondent + (1|qIdx) + (1|subIdx)**" will be the most plausible... ##

\### brm() parameter estimation results

\# Multilevel Hyperparameters:

\#	~qIdx (Number of levels: 18)

\#	Estimate Est.Error l-95% CI u-95% CI Rhat Bulk_ESS Tail_ESS

\# sd(Intercept)	5.20	0.93	3.76	7.34 1.00	1157	2198

\# ~subIdx (Number of levels: 13)

\#	Estimate Est.Error l-95% CI u-95% CI Rhat Bulk_ESS Tail_ESS

\# sd(Intercept)	1.57	0.50	0.86	2.74 1.00	1433	1959

\# Regression Coefficients:

\#	Estimate Est.Error l-95% CI u-95% CI Rhat Bulk_ESS Tail_ESS

\# Intercept	18.63	1.30	16.02	21.24 1.00	1134	1625

\# RespondentGPT4.1	-5.34	1.88	-8.97	-1.61 1.00	2783	2400

\# RespondentGPT4o	-3.98	1.82	-7.56	-0.26 1.00	2900	2434

\# Further Distributional Parameters:

\#	Estimate Est.Error l-95% CI u-95% CI Rhat Bulk_ESS Tail_ESS

\# sigma	2.71	0.13	2.46	2.99 1.00	4996	3947

**Results of convergence in parameter estimations (4,000 iterations, 2,000 warmup, and 3 chains in MCMC; with the base of “Human”)**. The graphs show (from top to bottom) intercept, coefficient for GPT4.1, coefficient for GPT4o, SDs for random effects of “questions” and “respondents”, respectively. Left: parameter distributions. Right: trace plots.

# **Supporting Information 05**

\## The deviation in evaluation scores between humans (mean) and LLMs in all questions

Note: The x-axis shows each token in a question (denoting “*token number* _ *token* _ *question id*”). The y-axis shows the deviations: The positive values (pink) denote that the mean of humans’ scores was larger than LLMs’ scores, while the negative values (black) denote that LLMs’ scores were larger than humans’ scores. For each question, the upper and lower facets show the results in GPT-4o and GPT-4.1, respectively.
