# Supplementary material for: Attempts to investigate what words trivia experts focus on in quiz questions: Human-AI comparison through “LLM-as-a-judge” approach
Source: PLoS One. 2026 Sep 22;21(9):e0358332. doi: 10.1371/journal.pone.0358332 (PMC13596818; doi:10.1371/journal.pone.0358332)
Supplement: Supplementary file 2 [file pone.0358332.s003.docx]

# Response to editor(s)

We appreciate your careful checks of the previously submitted manuscript. In the revised manuscript, yellow highlighting indicates the sections with major revisions (e.g., where we have added a new discussion or explanation).

In this document, we respond to the journal requirements.

\## Requirement

1. Please ensure that your manuscript meets PLOS ONE's style requirements, including those for file naming. The PLOS ONE style templates can be found at

<https://journals.plos.org/plosone/s/file?id=wjVg/PLOSOne_formatting_sample_main_body.pdf> and

[https://journals.plos.org/plosone/s/file?id=ba62/PLOSOne_formatting_sample_title_authors_affiliations.pdf](https://track.editorialmanager.com/CL0/https:%2F%2Fjournals.plos.org%2Fplosone%2Fs%2Ffile%3Fid=ba62%2FPLOSOne_formatting_sample_title_authors_affiliations.pdf/1/010f019be98c4ea2-533daa8d-6171-452b-9897-e7dbdd62830b-000000/S3Aj2OzmbsQE8NBw4cdxFozWW7vyKnYRRbKMySYAuvQ=247)

**Response**

According to the above URLs, we have checked the format of manuscript such as the citation style.

\## Requirement

2. Please note that your Data Availability Statement is currently missing the repository name and/or the DOI/accession number of each dataset. If your manuscript is accepted for publication, you will be asked to provide these details on a very short timeline. We therefore suggest that you provide this information now, though we will not hold up the peer review process if you are unable.

**Response**

In the submission system, we report the statements below:

- All prompt and code related to the system are available from GitHub platform:

<https://github.com/bravebird0914/llm-quiz-attribution>

- All data and code related to the behavioral experiment are available from OSF platform:

<https://osf.io/hvfsn/overview?view_only=fb2037eaaaf04cbea271a207dbc84f7a>

\## Requirements

3. We note that the figures contain text or data that is not in English. Please note that PLOS is an English-language publisher. Please replace it with an English-language version of your figures.

4. Thank you for providing your underlying data as Supporting Information.

We note that the data set contains text or data that is not in English. Please note that PLOS is an English-language publisher, so we require data sets to be provided in English as well. Please upload an English-language version of your data set.

**Response**

We have made every effort to provide materials with English versions. For example, we have provided English translations for the 18 questions we used in Supporting information and included the full English-translated text of each question in Fig 6.

However, it will be impossible to completely translate Japanese texts into English text word by word. All question texts in the database (2,480 questions in total) are all written by Japanese. Also, we had to input the system prompts in Japanese. It is almost impossible for us to translate all texts into English keeping the Japanese original meanings and nuances. We would be grateful for your understanding.

\## Requirement

5. Please include captions for your Supporting Information files at the end of your manuscript, and update any in-text citations to match accordingly. Please see our Supporting Information guidelines for more information: <http://journals.plos.org/plosone/s/supporting-information>.

**Response**

According to the above URL and the following format
( <https://journals.plos.org/plosone/s/file?id=wjVg/PLOSOne_formatting_sample_main_body.pdf> ),
we have added “Supporting information” section in the end of the main text, and have also revised in-text citations with square brackets instead of round parentheses.

Finally, thank you so much again for your editorial checks.

Masaru Shirasuna and Yuto Yoshida

# Response to reviewer 01

We appreciate your careful review of the previously submitted manuscript. We found the comments reasonable and helpful in improving our manuscript. We respect your comments and have attempted to respond to them below.

In the revised manuscript, yellow highlighting indicates the sections with major revisions (e.g., where we have added a new discussion or explanation).

\## Comment

1. The primary limitation of the study is its highly restricted experimental design, involving only 11 participants and 18 questions. Although framing the results as “preliminary findings” is reasonable, drawing broad claims about the superiority of human intelligence over LLMs from such a limited, task-specific dataset is scientifically risky. Moreover, the exclusive use of highly experienced trivia experts—who are likely familiar with the examined word–answer associations—suggests that the observed effects may reflect domain-specific, learned heuristics rather than general human intelligence. Accordingly, the study would be more appropriately framed as an investigation of expert pattern sensitivity. To strengthen its validity, the authors should either expand the sample size or clearly reposition the work as an exploratory, case-based analysis rather than definitive evidence.

**Response**

Thank you for your suggestions. As you pointed out, our sample size was small. So, we consider that we should not argue our findings strongly. To tone down our conclusions, we repositioned the current study as an exploratory, case-based analysis, rather than definitive and generalizable evidence, throughout our manuscript (e.g., emphasizing the scope in Introduction; avoiding definitive descriptions; using the word “experts” as possible instead of “humans”). In addition, we explicitly reported the problem about small sample size as one of the limitations of this study (due to our research resources and trivia experts’ circumstances, it was highly difficult to increase the sample size).

\## P8 L164

Our focus is characteristics of trivia experts’ cognition, neither improving AI systems nor establishing new standards for LLM-as-a-judge approaches. We use LLMs as comparative analytical (rather than as normative) tools. The aim is to find some similarities/differences between humans and LLMs through comparison of their performance in quiz questions. Note that we position this study as an exploratory, case-based study. It is because, as described later, the sample size in the behavioral experiment was so small. Findings that are obtained in this study should be captured as non-definitive evidence (e.g., clarifying not general human cognitive processes but experts-specific tendencies).

\## P24 L557

The first limitation is the small data. As in cases of expert studies such as professional dancer or magicians (e.g., [26,27]), the population of trivia experts is so limited and it is highly difficult for them to participate in an experiment (Especially in quizzes, most quiz players have their primary job outside of quizzes, so it is sometimes difficult for them to devote their time to academic experiments). Due to the small sample size and the small number of questions in the behavioral experiment, the current results are just preliminary findings, and we should not overstate them. We just investigated trivia experts’ cognition, which cannot always generalize all humans. Therefore, we should capture our findings as results of case-based analyses. To strengthen robustness, further examinations are needed by increasing the sample size and the number of questions in the future.

\## Comment

2. There is a clear mismatch between ecological validity and the experimental design. Although the study targets “fast and accurate judgment,” the absence of time pressure and the use of slider-based scoring induce a slow, analytical process lasting 30–60 minutes. This raises uncertainty as to whether the experiment captures rapid, intuitive in-competition decisions or slower deliberative evaluations. A stronger theoretical justification or the inclusion of a time-pressured control condition would strengthen the study’s validity.

**Response**

Thank you for your comments. We consider that such a mismatch is controversial and another limitation of our study.

Actually, before the experiment of this study, we conducted a pilot study using a response time-based experiment (i.e., participants were asked to push a button at the timing when they know the answer, which was more similar to an actual buzzer-quiz situation) to examine when and how trivia experts/novices would sequentially process information. As a result, we confirmed that the shape of graph of cumulative response time distribution was similar to that of the cumulative evaluation scores in this study. Based on this pilot study, and considering our aim to compare LLMs with experts, we chose the slider-based design in this study.

We have added such theoretical justifications in the Limitation section.

\## P25 L575

The third limitation is about experimental paradigms. One may argue that although this study targeted “fast and accurate judgments,” the absence of time pressure and the use of slider-based scoring in the experiment might induce participants’ slow and analytical processes, so it is unclear whether the experiment captured faster intuitive decisions or slower deliberative evaluations. Such a mismatch between ecological validity and experimental design may not be able to perfectly solve within the current study. However, our preliminary study (and also [8]) conducted response time-based (not slider-based) experiments to investigate the point at which trivia experts/novices could make correct inferences in buzzer quizzes. Then, we confirmed that the shape of graph for cumulative distributions of response time was highly similar to that of evaluation scores in the current study (convex upward). Based on such confirmations, we chose the slider-based design to align task formats between humans and LLMs.

\###

\# For reference: Although the questions used were not identical to those in this study, a similar experiment based on reaction time was conducted by [Shirasuna (2024](https://osf.io/preprints/psyarxiv/8q9u3_v1)) (a similar curve was observed in cumulative reaction time distributions of experts; Figure 6).

\## Comment

3. Another limitation concerns the restrictive prompt design and parameter settings used for the LLMs. Fixing the Temperature at 0.1 may have constrained probabilistic and intuitive behaviour, and limiting the analysis to GPT-4o and GPT-4.1 reduces generalisability across LLM architectures. Including a sensitivity analysis with different temperature values, prompt configurations, and additional model families would strengthen the methodological robustness.

**Response**

Thank you for your comment. According to your comment, we additionally manipulated prompts of a role and values of temperature parameter using GPT-4.1. Unfortunately, the official support for GPT-4o had ended when we conducted this additional analysis (Feb 18-28 2026), so we had to use only GPT-4.1.

Specifically, we excluded “You are a trivia expert” from the prompt and simply provided “Accurately evaluate the importance of the given tokens.” (No-prompt version). This allowed us to confirm that LLMs’ performance was driven by an inference ability by LLM itself rather than by a role prompt. We also tried to range values of temperature to a system default, 0.8 (default-temperature version). This could represent more randomness of LLMs’ outputs. In sum, we set four conditions in total: Providing prompt and 0.1 temperature (reported in the main text); Providing prompt and default temperature; Providing no-prompt and 0.1 temperature; and Providing no-prompt and default temperature.

For each condition, we ran ten times and calculated the averages of each token’s evaluation score in the ten runs (note that the reason for the number of runs, ten, was that approximately ten players compete quizzes in one round in many actual buzzer-quiz competitions). As shown in the following figure, LLMs’ performance did not so much differ regardless of these manipulations. Thus, we consider that we could confirm methodological robustness.

\## P12 L261

Note that to confirm the robustness of LLMs’ performance, we additionally manipulated the role prompt (Prompt and No-prompt) and temperature parameter (0.1 and system default). Specifically, we tried to prompt LLMs without “You are a trivia expert” to confirm that LLMs’ performance was driven by an inference ability by LLM itself rather than by a role prompt. We also tried to range values of temperature to a default, 0.8, which could represent more randomness of LLMs’ outputs. Regardless of these manipulations, LLMs’ performance remained almost unchanged (see Supporting information 2).

(cont’d)

\## supporting information (p. 21)

**Results of LLMs’ robustness check across four conditions**. The x-axis shows the token number (i.e., when the word/phrase was presented in a question). The y-axis shows the cumulative evaluation scores by LLMs. We conducted the following four conditions: Providing prompt and 0.1 temperature; Providing prompt and default temperature; Providing no-prompt and 0.1 temperature; and Providing no-prompt and default temperature. Each panel denotes each question, and the facet title in the gray bar represents the correct answer of the question.

\## Comment

4. The use of the term “solving” in the title is potentially misleading, as participants do not solve the questions but instead assign importance scores to words given both the question and the correct answer. The study focuses on word-level evaluation and attribution rather than problem solving.

**Response**

According to your suggestion, we deleted the term “solving” from the short title. The revised short title is “Comparison between trivia experts and LLMs in quiz questions.”

In addition, basically we have removed the term “solving/solve” from the manuscript.

\## Comment

5. The manuscript’s structure would benefit from refinement. Combining results and interpretation in a single “Results and Discussion” section makes it difficult to distinguish objective findings from commentary; separating these sections would improve clarity. The absence of a distinct “Conclusion” section should also be reconsidered in light of standard PLOS ONE format. In addition, sample-size limitations should be addressed more explicitly in a dedicated “Limitations” section, and clearer recommendations for future work would strengthen the contribution.

**Response**

According to your suggestion, we divided the “Results and discussion” section into the “Results” and “Discussion” sections. We have moved descriptions of implications/suggestions to the new Discussion section. In addition, we newly added the “Limitation” section in the General discussion section, and again clearly described limitations in our study such as the small sample size.

\## P22 L518

4.	Discussion

The results of overall performance imply that humans often focus on various words/phrases that LLMs may overlook. And the results of specific performance suggest that LLMs generally evaluate tokens that were strongly related to the correct answer as more important, regardless of quiz structures. Even if a token can uniquely identify the answer, LLMs sometimes judge it as less important if it seems to have weak associations with the answer.

Taken together, our case-based analyses reveal that trivia experts (i) will be able to focus more sensitively on words/phrases which can uniquely identify the answer; (ii) can make fast and accurate inductive inferences of what a generic concept was asked from a few types or examples; and (iii) often try to identify the answer from various words/phrases in a sentence even if they do not always seem to have a strong association with the answer. These tendencies may be the characteristics of human intelligence, which has much less knowledge than LLMs, in sequential information processing like buzzer quizzes.

\## P24 L556

5.1	Limitations

There are several limitations of this study. The first limitation is the small data. As in cases of expert studies such as professional dancer or magicians (e.g., [26,27]), the population of trivia experts is so limited and it is highly difficult for them to participate in an experiment (Especially in quizzes, most quiz players have their primary job outside of quizzes, so it is sometimes difficult for them to devote their time to academic experiments). Due to the small sample size and the small number of questions in the behavioral experiment, the current results are just preliminary findings, and we should not overstate them. We just investigated trivia experts’ cognition, which cannot always generalize all humans. Therefore, we should capture our findings as results of case-based analyses. To strengthen robustness, further examinations are needed by increasing the sample size and the number of questions in the future.

\## Comment

6. The citation format used throughout the manuscript should also be revised for consistency with the journal’s style guidelines.

**Response**

According to your suggestion, we revised the citation format throughout the manuscript, such as revising round parentheses into square brackets for in-text citations, and revising the References section according to “Formatting references” <https://journals.plos.org/plosone/s/submission-guidelines#loc-references>

\## Comment

7. The phrase “It is suggested that …” (p. 24) relies on a vague passive construction with an unspecified agent, which weakens the claim. “Sequentially” and “gradually” are partly redundant here; one of them could be removed for clarity (p. 26).

**Response**

Thank you for your suggestions. According to your suggestion, we revised the descriptions:

\## P23 L528

These tendencies may be the characteristics of human intelligence, which has much less knowledge than LLMs, in sequential information processing like buzzer quizzes.

\## P24 L548

By comparing LLMs’ and humans’ evaluations, we found that both humans and LLMs could gradually accumulate evidence for the correct answer, …

Finally, thank you again for your kind and constructive review of our manuscript.

Anonymized authors

# Response to reviewer 02

We appreciate your careful review of the previously submitted manuscript. We found the comments reasonable and helpful in improving our manuscript. We respect your comments and have attempted to respond to them below.

According to your summarization “**In conclusion, the study presents a systematic approach to a well-defined problem and reports several interesting findings. Addressing the points above—particularly by moderating the interpretive claims and clarifying methodological limitations related to human cognition—would substantially enhance the robustness and interpretability of the manuscript**”, we revised the manuscript trying to recognize several limitations and tone down our findings. Due to the small sample size and the limited material, we have repositioned this study as an exploratory as an exploratory analysis rather than definitive evidence.

In the revised manuscript, yellow highlighting indicates the sections with major revisions (e.g., where we have added a new discussion or explanation).

\## Comment

In particular, claims in the Results and Discussion sections suggesting that humans are “superior” to LLMs should be softened, given the indirect nature of the measurement approach. The findings convincingly demonstrate that humans and LLMs employ different strategies for accumulating and evaluating clues in trivia questions; however, interpreting these differences as direct evidence of superiority remains debatable within the current experimental framework.

**Response**

Thank you for your insightful suggestions. To avoid overstate our results, we generally deleted the term “superior/superiority” from the manuscript. For example:

\## P8 L166

The aim is to find some similarities/differences between humans and LLMs through comparison of their performance in quiz questions.

(original: We aim to find some similarities/differences between human and artificial intelligence, particularly some superiorities of human intelligence, … through comparison of their performance in trivia quiz questions)

\## P20 L473

… experts may be able to faster and more accurately capture the word/phrase at which the answer can be uniquely identified, than LLMs

(original: humans will be superior to LLMs in fast and accurately judging the word/phrase at which the answer can be uniquely identified)

\## Comment

From a methodological perspective, an important consideration is that human participants were required to evaluate pre-defined, tokenized word or phrase units. In real-world trivia or buzzer quiz settings, humans are unlikely to process questions strictly on a word-by-word basis; instead, they tend to rely on more holistic and contextual interpretations. As such, the task design may impose an artificial constraint on human cognitive processes, reflecting a form of “human evaluation adapted to an LLM-oriented format” rather than a direct measurement of natural human cognition. Explicitly acknowledging this limitation would strengthen the conceptual clarity of the study.

**Response**

Thank you so much for your comment. We agree with your insights and admit this issue as a limitation.

On the other hand, actual quiz players say that they study/learn quiz questions focusing on not only holistic context but also word-by-word level (i.e., considering which word can be critical to identify the correct answer) to respond as fast and accurately as possible; even in actual buzzer-quiz competition situations, they sequentially pay attention to each word while hearing a question text (Izawa, 2021; Shirasuna & Kosaka, 2024). So, we consider that our experiment might be an LLM-oriented format but could also reflect somewhat actual trivia experts’ processes.

We have added such discussion as one of the limitations.

\## P25 L586

Another related methodological issue should also be pointed out. In our experiments, participants were required to evaluate pre-defined, tokenized words/phrase units. In contrast, it is considered that in actual buzzer quizzes, humans are unlikely to process questions strictly on a word-by-word basis; instead, they may rely on more holistic and contextual interpretations. Thus, our experimental design might impose an artificial constraint on human cognitive processes and reflect a form of “human evaluation adapted to an LLM-oriented format” rather than natural human cognition. Experimental designs will still be controversial. However, human trivia experts often study quiz questions considering which word will be critical to faster identify the correct answer, focusing on each word/phrase level even in actual buzzer quizzes [6]. Therefore, we believe that actual trivia experts’ processes could be more or less reflected in our experiment.

\## Comment

In addition, within the LLM-as-a-judge framework, it would be helpful to emphasize more clearly that LLMs are used as comparative analytical tools rather than as normative or ground-truth judges.

**Response**

Thank you for your suggestions. We emphasized this point in the Study outline and Limitation sections.

\## P8 L164

Our focus is characteristics of trivia experts’ cognition, neither improving AI systems nor establishing new standards for LLM-as-a-judge approaches. We use LLMs as comparative analytical (rather than as normative) tools.

\## P24 L569

Relatedly, we should emphasize more clearly that within the LLM-as-a-judge framework, LLMs were used in this study as comparative analytical tools rather than as normative or ground-truth judges. This study just explored the characteristics of trivia experts’ cognition in quiz questions through LLMs.

\## Comment

Finally, some implementation-level details related to tokenization and importance scoring may be overly detailed for the main text and could be moved to supplementary materials to improve readability.

**Response**

We remain the outline of system descriptions in the main text and have moved the details such as about tokenization scoring to the Supporting information. The section 2.1 “Theoretical study: Implementing “quiz question processing system”’ originally had 6 pages but was shortened to 3.5 pages (P9-12).

Finally, thank you again for your kind and constructive review of our manuscript.

Anonymized authors
