# Supplementary material for: Attempts to investigate what words trivia experts focus on in quiz questions: Human-AI comparison through “LLM-as-a-judge” approach
Source: PLoS One. 2026 Sep 22;21(9):e0358332. doi: 10.1371/journal.pone.0358332 (PMC13596818; doi:10.1371/journal.pone.0358332)
Supplement: Supplementary file 3 [file pone.0358332.s004.docx]

# Response to reviewer 01

We appreciate your careful review of the previously submitted manuscript. We found the comments reasonable and helpful in improving our manuscript. We respect your comments and have attempted to respond to them below.

In the revised manuscript, yellow highlighting indicates the sections with major revisions (e.g., where we have added a new discussion or explanation).

\## Comment

The authors reference their pilot study [8] to justify the slider-based design. However, the fact that both paradigms produce "convex upward" cumulative curves does not constitute strong evidence of convergent validity, as this is a generic shape that most cumulative distributions would exhibit regardless of the underlying cognitive process. The term "confirmed" (L580–584) should be softened to reflect this.

**Response**

According to your suggestion, first, we removed the statements based on the shape of the graph (such as “convex upward”). Then, we cited the reports and discussions by actual trivia experts as evidence supporting the validity of the experimental design. (On the other hand, however, we think that the mismatch between ecological validity and experimental design that you pointed out cannot be perfectly solved within the current study, which is a limitation of this study.)

\## P28 L583

The third limitation is about experimental paradigms. One may argue that although this study targeted “fast and accurate judgments,” the absence of time pressure and the use of slider-based scoring in the experiment might induce participants’ slow and analytical processes. Thus, it is unclear whether the experiment captured faster intuitive decisions or slower deliberative evaluations. Such a mismatch between ecological validity and experimental design may not be able to perfectly solve within the current study. That said, to some extent, this design might be able to be reflected trivia experts’ thinking processes in actual competitive quiz situations. In fact, according to actual trivia experts (e.g., [6], [10]), even in competitive situations where real-time and instantaneous judgments are required, players identify alternative answers into a single correct answer based on information (word/phrase) that is gradually revealed. They also reported that they sometimes feel the processes from obtaining information to considering the answer as extremely slow, even like slow motion. Therefore, we believe that although it was not competitive quiz situations, our behavioral experiment where participants evaluated the importance of each individual word/phrase was suitable to some extent, to experimentally investigate their thinking processes.

\## Comment

The additional sensitivity analysis regarding temperature and prompt configurations is appreciated and adequately addresses those aspects of the original concern. However, the issue of generalizability across LLM architectures remains unaddressed. The analysis is still limited to a single model family (GPT). While the discontinuation of GPT-4o support is understandable, testing at least one model from a different architecture (e.g., Claude, Gemini, or an open-source model such as Llama) would have strengthened the claim of robustness. The authors should at minimum acknowledge this as a limitation.

**Response**

According to your suggestion, we have added this issue to the Limitation section.

\## P29 L598

The fourth limitation is the generalizability across LLM architectures. To confirm the robustness of LLM’s evaluations, we manipulated some factors such as the temperature parameter and prompts (see Method section). However, our analysis was still limited to a single model family (GPT). It is more desirable to test at least one model from a different architecture such as Claude, Gemini, or an open-source model such as Llama (note that, unfortunately, the official support for GPT-4o ended after our experiments). By using these models, we will be able to strengthen the robustness of our results.

Finally, thank you again for your kind and constructive review of our manuscript.

*Anonymized authors*

# Response to reviewer 02

We appreciate your careful review of the previously submitted manuscript. We found the comments reasonable and helpful in improving our manuscript. We respect your comments and have attempted to respond to them below.

In the revised manuscript, yellow highlighting indicates the sections with major revisions (e.g., where we have added a new discussion or explanation).

\## Comment

First, although the authors have partially moderated their conclusions, some statements still suggest that human experts are “better” than LLMs. Given the indirect nature of the measurement and the experimental design, such claims should be further softened and consistently expressed using more cautious language (e.g., “suggest,” “may indicate,” or “are consistent with”).

**Response**

According to your suggestion, we have revised some descriptions. For example:

\## abstract

It may be indicated that trivia experts can make inductive inferences faster (e.g., finding a common concept even from few items).

\## P21 L447

Compared to LLMs, experts might focus more sensitively on a word/phrase at which the correct answer is uniquely identified.

\## P26 L527

Taken together, our case-based analyses suggest that trivia experts may (i) be able to focus more sensitively on words/phrases which can uniquely identify the answer; ….

\## Comment

Second, while the positioning of the LLM-as-a-judge framework has improved, in some parts of the manuscript LLM outputs still appear to function implicitly as a benchmark. It would strengthen the paper to more clearly and consistently emphasize that LLMs are used solely as comparative tools, and that their validity as evaluators remains an open methodological question.

**Response**

According to your suggestions, we deleted the word “benchmark(s)” and used “comparative tool(s)” throughout the revised manuscript. In addition, we emphasized this issue in the Limitation section.

\## P28 L577

Relatedly, we should emphasize clearly that within the LLM-as-a-judge framework, this study used LLMs just as comparative analytical tools rather than as normative or ground-truth judges. We explored the characteristics of trivia experts’ cognition in quiz questions through LLMs, and LLMs’ validity as evaluators remains an open methodological question. That being said, we expect that this study will be the first step to highlight human intelligence to correctly and accurately respond to a question from limited information.

\## Comment

Third, although the limitations of token-level evaluation are acknowledged, it would be beneficial to more explicitly clarify that the task reflects a structured, post-hoc importance judgment rather than real-time decision-making in natural quiz settings. Making this distinction clearer would improve the interpretability of the findings and their ecological validity.

**Response**

Thank you for your comments. Regarding the mismatch between ecological validity and experimental design, we cited the reports and discussions by actual trivia experts as evidence supporting the validity of the experimental design. We think that based on them, our experimental design could reflect the actual competitive and real-time quiz situations to some extent. That said, however, we also think that this mismatch cannot be perfectly solved within the current study, which is a limitation of this study.

\## P28 L583

The third limitation is about experimental paradigms. One may argue that although this study targeted “fast and accurate judgments,” the absence of time pressure and the use of slider-based scoring in the experiment might induce participants’ slow and analytical processes. Thus, it is unclear whether the experiment captured faster intuitive decisions or slower deliberative evaluations. Such a mismatch between ecological validity and experimental design may not be able to perfectly solve within the current study. That said, to some extent, this design might be able to be reflected trivia experts’ thinking processes in actual competitive quiz situations. In fact, according to actual trivia experts (e.g., [6], [10]), even in competitive situations where real-time and instantaneous judgments are required, players identify alternative answers into a single correct answer based on information (word/phrase) that is gradually revealed. They also reported that they sometimes feel the processes from obtaining information to considering the answer as extremely slow, even like slow motion. Therefore, we believe that although it was not competitive quiz situations, our behavioral experiment where participants evaluated the importance of each individual word/phrase was suitable to some extent, to experimentally investigate their thinking processes.

\## Comment

Finally, the validation of the proposed system remains limited to a brief qualitative inspection by two experts. While this may be acceptable within an exploratory framework, the implications of this limitation for the reliability of the results should be stated more explicitly.

**Response**

Thank you for your suggestions. We discussed this issue as one of the limitations.

\## P28 L571

The second limitation is the system validation. In the theoretical study, two quiz players briefly scanned our system’s outputs. This inspection may be acceptable within an exploratory framework, but it needs more rigorous, objective and quantitative validation to use LLM as a more reliable tool (see also [28]), such as scanning by more trivia experts and testing other models (see also below). Brief and qualitative checks in this study may affect the validity and applicability of our systems, and the generalizability of our findings. Therefore, further verification is required in the future.

Finally, thank you again for your kind and constructive review of our manuscript.

*Anonymized authors*
