# Supplementary material for: Attempts to investigate what words trivia experts focus on in quiz questions: Human-AI comparison through “LLM-as-a-judge” approach
Source: PLoS One. 2026 Sep 22;21(9):e0358332. doi: 10.1371/journal.pone.0358332 (PMC13596818; doi:10.1371/journal.pone.0358332)
Supplement: Supplementary file 4 [file pone.0358332.s005.docx]

# Response to reviewer 01

We appreciate your careful review of the previously submitted manuscript. We found the comments reasonable and helpful in improving our manuscript. We respect your comments and have attempted to respond to them below.

In the revised manuscript, yellow highlighting indicates the sections with revisions (e.g., where we have edited English descriptions and added a new explanation).

\## Comment

-The manuscript contains grammatical errors and awkward constructions in several places (e.g., L148, L358, L587–589, and similar instances throughout the text). A thorough language edit by a professional editing service or a fluent English speaker is recommended.

**Response**

We had our English text edited by an editing service *Editage* ([www.editage.jp](http://www.editage.jp)). Native English speakers have checked our manuscript in terms of the grammar and usage. We show examples of edited descriptions in our manuscript and the editing certificate from Editage.

```
## e.g. (Note: In the revised manuscript, we also have yellow-highlighted on other revisions.)
## (previous L148)
```

Of course, to conduct such approaches, LLMs need to be tuned for answering quiz questions.

\## (revised L148)

To apply such an approach, LLMs need to be tuned for answering quiz questions.

\## (previous L358)

To examine trivia experts’ considerations in quiz questions, we compared human’s evaluation scores to system's ones. Specifically, we calculated cumulative their evaluation scores.

\## (revised L358)

To examine how trivia experts weighed information in quiz questions, we compared humans’ evaluation scores with those of the system. Specifically, we calculated their cumulative evaluation scores.

\## (previous L587)

Such a mismatch between ecological validity and experimental design may not be able to perfectly solve within the current study.

\## (revised L591)

This mismatch between ecological validity and experimental design cannot be fully resolved within the present study.

\## certificate of editing (author names anonymized)

\## Comment

-The phrase "two latest LLMs" (Abstract, L217, L548) is no longer accurate, as newer models have since been released. A more time-robust wording such as "two widely used LLMs" is recommended.

**Response**

According to your suggestion, we have revised these descriptions.

\## L217

We used the two widely used LLMs, GPT-4o and GPT-4.1, with identical prompt designs and hyperparameters to ensure comparability of outputs.

\## L552

… evaluates the importance of each token using two widely used LLMs, GPT-4o and GPT-4.1.

\## Comment

-The image quality of Fig 2 is insufficient (blurry text with visible compression artifacts); a higher-resolution version should be provided.

**Response**

We revised Fig 2 with a higher resolution.

Finally, thank you again for your kind and constructive review of our manuscript.

*Anonymized authors*
