# Supplementary material for: Attempts to investigate what words trivia experts focus on in quiz questions: Human-AI comparison through “LLM-as-a-judge” approach
Source: PLoS One. 2026 Sep 22;21(9):e0358332. doi: 10.1371/journal.pone.0358332 (PMC13596818; doi:10.1371/journal.pone.0358332)
Supplement: Peer review history

# Peer review: Decision Letter 0

**DOI:** 10.1371/journal.pone.0358332.r001

**Authors:** Musa Aydin

23 Jan 2026

Dear Dr. Shirasuna,

Thank you for submitting your manuscript to PLOS ONE. After careful consideration, we feel that it has merit but does not fully meet PLOS ONE’s publication criteria as it currently stands. Therefore, we invite you to submit a revised version of the manuscript that addresses the points raised during the review process.

Please submit your revised manuscript by Mar 09 2026 11:59PM. If you will need more time than this to complete your revisions, please reply to this message or contact the journal office at plosone@plos.org. When you're ready to submit your revision, log on to https://www.editorialmanager.com/pone/ and select the 'Submissions Needing Revision' folder to locate your manuscript file.

- A letter that responds to each point raised by the academic editor and reviewer(s). You should upload this letter as a separate file labeled 'Response to Reviewers'.
- A marked-up copy of your manuscript that highlights changes made to the original version. You should upload this as a separate file labeled 'Revised Manuscript with Track Changes'.
- An unmarked version of your revised paper without tracked changes. You should upload this as a separate file labeled 'Manuscript'.

If you would like to make changes to your financial disclosure, please include your updated statement in your cover letter. Guidelines for resubmitting your figure files are available below the reviewer comments at the end of this letter.

If applicable, we recommend that you deposit your laboratory protocols in protocols.io to enhance the reproducibility of your results. Protocols.io assigns your protocol its own identifier (DOI) so that it can be cited independently in the future. For instructions see: https://journals.plos.org/plosone/s/submission-guidelines#loc-laboratory-protocols. Additionally, PLOS ONE offers an option for publishing peer-reviewed Lab Protocol articles, which describe protocols hosted on protocols.io. Read more information on sharing protocols at https://plos.org/protocols?utm_medium=editorial-email&utm_source=authorletters&utm_campaign=protocols.

We look forward to receiving your revised manuscript.

Kind regards,

Musa Aydin, Ph.D.

Academic Editor

PLOS One

Journal Requirements:

When submitting your revision, we need you to address these additional requirements.

1. Please ensure that your manuscript meets PLOS ONE's style requirements, including those for file naming. The PLOS ONE style templates can be found at

https://journals.plos.org/plosone/s/file?id=wjVg/PLOSOne_formatting_sample_main_body.pdf and

https://journals.plos.org/plosone/s/file?id=ba62/PLOSOne_formatting_sample_title_authors_affiliations.pdf

2. Please note that your Data Availability Statement is currently missing the repository name and/or the DOI/accession number of each dataset. If your manuscript is accepted for publication, you will be asked to provide these details on a very short timeline. We therefore suggest that you provide this information now, though we will not hold up the peer review process if you are unable.

3. We note that the figures contain text or data that is not in English. Please note that PLOS is an English-language publisher. Please replace it with an English-language version of your figures.

4. Thank you for providing your underlying data as Supporting Information.

We note that the data set contains text or data that is not in English. Please note that PLOS is an English-language publisher, so we require data sets to be provided in English as well. Please upload an English-language version of your data set.

This will also allow us to determine if your data follows PLOS standards per our Data Availability policy here: https://journals.plos.org/plosone/s/data-availability

5. Please include captions for your Supporting Information files at the end of your manuscript, and update any in-text citations to match accordingly. Please see our Supporting Information guidelines for more information: http://journals.plos.org/plosone/s/supporting-information.

If the reviewer comments include a recommendation to cite specific previously published works, please review and evaluate these publications to determine whether they are relevant and should be cited. There is no requirement to cite these works unless the editor has indicated otherwise.

[Note: HTML markup is below. Please do not edit.]

Reviewers' comments:

Reviewer's Responses to Questions

**Comments to the Author**

1. Is the manuscript technically sound, and do the data support the conclusions?

Reviewer #1: Partly

Reviewer #2: Yes

**********

2. Has the statistical analysis been performed appropriately and rigorously?

Reviewer #1: I Don't Know

Reviewer #2: Yes

**********

3. Have the authors made all data underlying the findings in their manuscript fully available?

The PLOS Data policy

Reviewer #1: Yes

Reviewer #2: Yes

**********

4. Is the manuscript presented in an intelligible fashion and written in standard English?

Reviewer #1: Yes

Reviewer #2: Yes

**********

Reviewer #1: This study compares decision-making under limited information between human experts and Large Language Models (LLMs) using data from “abc” quiz competitions in Japan. By treating LLMs as a comparative reference, the authors quantitatively assess how humans converge on correct answers from sparse cues. The work is valuable in highlighting differences between human and LLM behaviour—particularly in inductive reasoning and structural awareness—using AUC-based evidence. However, certain methodological constraints and issues related to the paper’s titling moderately limit its interpretative scope and generalisability. From a technical standpoint, the multi-stage Japanese-specific tokenisation pipeline and the use of AUC and Bayesian multilevel modelling strengthen the analysis, while framing LLMs as an evaluation reference represents an original contribution.

1. The primary limitation of the study is its highly restricted experimental design, involving only 11 participants and 18 questions. Although framing the results as “preliminary findings” is reasonable, drawing broad claims about the superiority of human intelligence over LLMs from such a limited, task-specific dataset is scientifically risky. Moreover, the exclusive use of highly experienced trivia experts—who are likely familiar with the examined word–answer associations—suggests that the observed effects may reflect domain-specific, learned heuristics rather than general human intelligence. Accordingly, the study would be more appropriately framed as an investigation of expert pattern sensitivity. To strengthen its validity, the authors should either expand the sample size or clearly reposition the work as an exploratory, case-based analysis rather than definitive evidence.

2. There is a clear mismatch between ecological validity and the experimental design. Although the study targets “fast and accurate judgment,” the absence of time pressure and the use of slider-based scoring induce a slow, analytical process lasting 30–60 minutes. This raises uncertainty as to whether the experiment captures rapid, intuitive in-competition decisions or slower deliberative evaluations. A stronger theoretical justification or the inclusion of a time-pressured control condition would strengthen the study’s validity.

3. Another limitation concerns the restrictive prompt design and parameter settings used for the LLMs. Fixing the Temperature at 0.1 may have constrained probabilistic and intuitive behaviour, and limiting the analysis to GPT-4o and GPT-4.1 reduces generalisability across LLM architectures. Including a sensitivity analysis with different temperature values, prompt configurations, and additional model families would strengthen the methodological robustness.

4. The use of the term “solving” in the title is potentially misleading, as participants do not solve the questions but instead assign importance scores to words given both the question and the correct answer. The study focuses on word-level evaluation and attribution rather than problem solving.

5. The manuscript’s structure would benefit from refinement. Combining results and interpretation in a single “Results and Discussion” section makes it difficult to distinguish objective findings from commentary; separating these sections would improve clarity. The absence of a distinct “Conclusion” section should also be reconsidered in light of standard PLOS ONE format. In addition, sample-size limitations should be addressed more explicitly in a dedicated “Limitations” section, and clearer recommendations for future work would strengthen the contribution.

6. The citation format used throughout the manuscript should also be revised for consistency with the journal’s style guidelines.

7. The phrase “It is suggested that …” (p. 24) relies on a vague passive construction with an unspecified agent, which weakens the claim. “Sequentially” and “gradually” are partly redundant here; one of them could be removed for clarity (p. 26).

Reviewer #2: This study addresses an interesting and timely problem by comparing how human trivia experts and large language models (LLMs) evaluate trivia questions under conditions of limited information. Overall, the manuscript is well written, the experimental procedure is presented transparently, and the methods are described in sufficient detail. The reader can clearly understand what was done and how the study was conducted.

That said, several conceptual and methodological points would benefit from a more cautious treatment. In particular, claims in the Results and Discussion sections suggesting that humans are “superior” to LLMs should be softened, given the indirect nature of the measurement approach. The findings convincingly demonstrate that humans and LLMs employ different strategies for accumulating and evaluating clues in trivia questions; however, interpreting these differences as direct evidence of superiority remains debatable within the current experimental framework.

From a methodological perspective, an important consideration is that human participants were required to evaluate pre-defined, tokenized word or phrase units. In real-world trivia or buzzer quiz settings, humans are unlikely to process questions strictly on a word-by-word basis; instead, they tend to rely on more holistic and contextual interpretations. As such, the task design may impose an artificial constraint on human cognitive processes, reflecting a form of “human evaluation adapted to an LLM-oriented format” rather than a direct measurement of natural human cognition. Explicitly acknowledging this limitation would strengthen the conceptual clarity of the study.

In addition, within the LLM-as-a-judge framework, it would be helpful to emphasize more clearly that LLMs are used as comparative analytical tools rather than as normative or ground-truth judges. Finally, some implementation-level details related to tokenization and importance scoring may be overly detailed for the main text and could be moved to supplementary materials to improve readability.

In conclusion, the study presents a systematic approach to a well-defined problem and reports several interesting findings. Addressing the points above—particularly by moderating the interpretive claims and clarifying methodological limitations related to human cognition—would substantially enhance the robustness and interpretability of the manuscript.

**********

what does this mean?). If published, this will include your full peer review and any attached files.

If you choose “no”, your identity will remain anonymous but your review may still be made public.

**Do you want your identity to be public for this peer review?**  For information about this choice, including consent withdrawal, please see our Privacy Policy

Reviewer #1: No

Reviewer #2: No

**********

[NOTE: If reviewer comments were submitted as an attachment file, they will be attached to this email and accessible via the submission site. Please log into your account, locate the manuscript record, and check for the action link "View Attachments". If this link does not appear, there are no attachment files.]

To ensure your figures meet our technical requirements, please review our figure guidelines: https://journals.plos.org/plosone/s/figures

You may also use PLOS’s free figure tool, NAAS, to help you prepare publication quality figures: https://journals.plos.org/plosone/s/figures#loc-tools-for-figure-preparation.

NAAS will assess whether your figures meet our technical requirements by comparing each figure against our figure specifications.

# Author comment: Author response to Decision Letter 1

**DOI:** 10.1371/journal.pone.0358332.r002

6 Mar 2026

Please see the attached file "Response to Reviewers" (file name: 260305v02HumanAW_ResponsePone.docx).

Attachment Submitted filename: 260305v02HumanAW_ResponsePone.docx

# Peer review: Decision Letter 1

**DOI:** 10.1371/journal.pone.0358332.r003

**Authors:** Musa Aydin

23 Apr 2026

Dear Dr. Shirasuna,

Thank you for submitting your manuscript to PLOS ONE. After careful consideration, we feel that it has merit but does not fully meet PLOS ONE’s publication criteria as it currently stands. Therefore, we invite you to submit a revised version of the manuscript that addresses the points raised during the review process.

Please submit your revised manuscript by Jun 07 2026 11:59PM. If you will need more time than this to complete your revisions, please reply to this message or contact the journal office at plosone@plos.org. When you're ready to submit your revision, log on to https://www.editorialmanager.com/pone/ and select the 'Submissions Needing Revision' folder to locate your manuscript file.

- A letter that responds to each point raised by the academic editor and reviewer(s). You should upload this letter as a separate file labeled 'Response to Reviewers'.
- A marked-up copy of your manuscript that highlights changes made to the original version. You should upload this as a separate file labeled 'Revised Manuscript with Track Changes'.
- An unmarked version of your revised paper without tracked changes. You should upload this as a separate file labeled 'Manuscript'.

If you would like to make changes to your financial disclosure, please include your updated statement in your cover letter. Guidelines for resubmitting your figure files are available below the reviewer comments at the end of this letter.

If applicable, we recommend that you deposit your laboratory protocols in protocols.io to enhance the reproducibility of your results. Protocols.io assigns your protocol its own identifier (DOI) so that it can be cited independently in the future. For instructions see: https://journals.plos.org/plosone/s/submission-guidelines#loc-laboratory-protocols. Additionally, PLOS ONE offers an option for publishing peer-reviewed Lab Protocol articles, which describe protocols hosted on protocols.io. Read more information on sharing protocols at https://plos.org/protocols?utm_medium=editorial-email&utm_source=authorletters&utm_campaign=protocols.

As the corresponding author, your ORCID iD is verified in the submission system and will appear in the published article. PLOS supports the use of ORCID, and we encourage all coauthors to register for an ORCID iD and use it as well. Please encourage your coauthors to verify their ORCID iD within the submission system before final acceptance, as unverified ORCID iDs will not appear in the published article. *Only*  the individual author can complete the verification step; PLOS staff *cannot*  verify ORCID iDs on behalf of authors.

We look forward to receiving your revised manuscript.

Kind regards,

Musa Aydin, Ph.D.

Academic Editor

PLOS One

**Journal Requirements:**

If the reviewer comments include a recommendation to cite specific previously published works, please review and evaluate these publications to determine whether they are relevant and should be cited. There is no requirement to cite these works unless the editor has indicated otherwise.

[Note: HTML markup is below. Please do not edit.]

Reviewers' comments:

Reviewer's Responses to Questions

**Comments to the Author**

Reviewer #1: (No Response)

Reviewer #2: (No Response)

**********

2. Is the manuscript technically sound, and do the data support the conclusions?

Reviewer #1: Partly

Reviewer #2: Partly

**********

3. Has the statistical analysis been performed appropriately and rigorously?

Reviewer #1: Yes

Reviewer #2: (No Response)

**********

4. Have the authors made all data underlying the findings in their manuscript fully available?

The PLOS Data policy

Reviewer #1: Yes

Reviewer #2: Yes

**********

5. Is the manuscript presented in an intelligible fashion and written in standard English?

Reviewer #1: Yes

Reviewer #2: Yes

**********

**Reviewer #1:** I thank the authors for their thorough revisions. The manuscript has improved substantially. Most of my original concerns have been adequately addressed. However, two points remain that require minor attention before acceptance.

Below I evaluate each response:

Comment 1: Adequately addressed.

Comment 2: The authors reference their pilot study [8] to justify the slider-based design. However, the fact that both paradigms produce "convex upward" cumulative curves does not constitute strong evidence of convergent validity, as this is a generic shape that most cumulative distributions would exhibit regardless of the underlying cognitive process. The term "confirmed" (L580–584) should be softened to reflect this.

Comment 3: The additional sensitivity analysis regarding temperature and prompt configurations is appreciated and adequately addresses those aspects of the original concern. However, the issue of generalizability across LLM architectures remains unaddressed. The analysis is still limited to a single model family (GPT). While the discontinuation of GPT-4o support is understandable, testing at least one model from a different architecture (e.g., Claude, Gemini, or an open-source model such as Llama) would have strengthened the claim of robustness. The authors should at minimum acknowledge this as a limitation.

Comments 4–7: Adequately addressed.

**Reviewer #2:**  I have carefully reviewed the revised version of the manuscript. The authors have made a clear and commendable effort to address the previous comments, and the manuscript has improved substantially in terms of both conceptual clarity and overall presentation. In particular, the more explicit positioning of the study as exploratory and case-based, the clearer discussion of the experimental limitations, and the improved framing of the LLM-as-a-judge approach as a comparative analytical tool rather than a normative ground truth are all positive developments. Overall, the revised version is more balanced and easier to interpret.

That said, a few minor but important issues remain to be addressed before the manuscript can be considered for acceptance. First, although the authors have partially moderated their conclusions, some statements still suggest that human experts are “better” than LLMs. Given the indirect nature of the measurement and the experimental design, such claims should be further softened and consistently expressed using more cautious language (e.g., “suggest,” “may indicate,” or “are consistent with”).

Second, while the positioning of the LLM-as-a-judge framework has improved, in some parts of the manuscript LLM outputs still appear to function implicitly as a benchmark. It would strengthen the paper to more clearly and consistently emphasize that LLMs are used solely as comparative tools, and that their validity as evaluators remains an open methodological question.

Third, although the limitations of token-level evaluation are acknowledged, it would be beneficial to more explicitly clarify that the task reflects a structured, post-hoc importance judgment rather than real-time decision-making in natural quiz settings. Making this distinction clearer would improve the interpretability of the findings and their ecological validity.

Finally, the validation of the proposed system remains limited to a brief qualitative inspection by two experts. While this may be acceptable within an exploratory framework, the implications of this limitation for the reliability of the results should be stated more explicitly.

Overall, the manuscript addresses an interesting and timely research question and presents a novel perspective on human–LLM comparison. The authors have made meaningful revisions, and the paper is now close to being publishable. Addressing the points above would further strengthen its clarity and rigor.

**********

what does this mean?). If published, this will include your full peer review and any attached files.

If you choose “no”, your identity will remain anonymous but your review may still be made public.

**Do you want your identity to be public for this peer review?**  For information about this choice, including consent withdrawal, please see our Privacy Policy

Reviewer #1: No

Reviewer #2: No

**********

[NOTE: If reviewer comments were submitted as an attachment file, they will be attached to this email and accessible via the submission site. Please log into your account, locate the manuscript record, and check for the action link "View Attachments". If this link does not appear, there are no attachment files.]

To ensure your figures meet our technical requirements, please review our figure guidelines: https://journals.plos.org/plosone/s/figures

You may also use PLOS’s free figure tool, NAAS, to help you prepare publication quality figures: https://journals.plos.org/plosone/s/figures#loc-tools-for-figure-preparation.

NAAS will assess whether your figures meet our technical requirements by comparing each figure against our figure specifications.

# Author comment: Author response to Decision Letter 2

**DOI:** 10.1371/journal.pone.0358332.r004

18 May 2026

Please see the Response to Reviewers "260518HumanAW_ResponsePone002.docx".

Attachment Submitted filename: 260518HumanAW_ResponsePone002.docx

# Peer review: Decision Letter 2

**DOI:** 10.1371/journal.pone.0358332.r005

**Authors:** Musa Aydin

4 Aug 2026

Dear Dr. Shirasuna,

Thank you for submitting your manuscript to PLOS ONE. After careful consideration, we feel that it has merit but does not fully meet PLOS ONE’s publication criteria as it currently stands. Therefore, we invite you to submit a revised version of the manuscript that addresses the points raised during the review process.

Please submit your revised manuscript by Sep 18 2026 11:59PM. If you will need more time than this to complete your revisions, please reply to this message or contact the journal office at plosone@plos.org. When you're ready to submit your revision, log on to https://www.editorialmanager.com/pone/ and select the 'Submissions Needing Revision' folder to locate your manuscript file.

- A letter that responds to each point raised by the academic editor and reviewer(s). You should upload this letter as a separate file labeled 'Response to Reviewers'.
- A marked-up copy of your manuscript that highlights changes made to the original version. You should upload this as a separate file labeled 'Revised Manuscript with Track Changes'.
- An unmarked version of your revised paper without tracked changes. You should upload this as a separate file labeled 'Manuscript'.

If you would like to make changes to your financial disclosure, please include your updated statement in your cover letter. Guidelines for resubmitting your figure files are available below the reviewer comments at the end of this letter.

If applicable, we recommend that you deposit your laboratory protocols in protocols.io to enhance the reproducibility of your results. Protocols.io assigns your protocol its own identifier (DOI) so that it can be cited independently in the future. For instructions see: https://journals.plos.org/plosone/s/submission-guidelines#loc-laboratory-protocols. Additionally, PLOS ONE offers an option for publishing peer-reviewed Lab Protocol articles, which describe protocols hosted on protocols.io. Read more information on sharing protocols at https://plos.org/protocols?utm_medium=editorial-email&utm_source=authorletters&utm_campaign=protocols.

As the corresponding author, your ORCID iD is verified in the submission system and will appear in the published article. PLOS supports the use of ORCID, and we encourage all coauthors to register for an ORCID iD and use it as well. Please encourage your coauthors to verify their ORCID iD within the submission system before final acceptance, as unverified ORCID iDs will not appear in the published article. *Only*  the individual author can complete the verification step; PLOS staff *cannot*  verify ORCID iDs on behalf of authors.

We look forward to receiving your revised manuscript.

Kind regards,

Musa Aydin, Ph.D.

Academic Editor

PLOS One

Journal Requirements:

If the reviewer comments include a recommendation to cite specific previously published works, please review and evaluate these publications to determine whether they are relevant and should be cited. There is no requirement to cite these works unless the editor has indicated otherwise.

Please review your reference list to ensure that it is complete and correct. If you have cited papers that have been retracted, please include the rationale for doing so in the manuscript text, or remove these references and replace them with relevant current references. Any changes to the reference list should be mentioned in the rebuttal letter that accompanies your revised manuscript. If you need to cite a retracted article, indicate the article’s retracted status in the References list and also include a citation and full reference for the retraction notice.

[Note: HTML markup is below. Please do not edit.]

Reviewers' comments:

Reviewer's Responses to Questions

**Comments to the Author**

Reviewer #1: (No Response)

Reviewer #2: All comments have been addressed

**********

2. Is the manuscript technically sound, and do the data support the conclusions?

Reviewer #1: Yes

Reviewer #2: Yes

**********

3. Has the statistical analysis been performed appropriately and rigorously?

Reviewer #1: Yes

Reviewer #2: Yes

**********

4. Have the authors made all data underlying the findings in their manuscript fully available?

The PLOS Data policy

Reviewer #1: Yes

Reviewer #2: Yes

**********

5. Is the manuscript presented in an intelligible fashion and written in standard English?

Reviewer #1: No

Reviewer #2: Yes

**********

Reviewer #1: I thank the authors for their careful revisions. My previous concerns have been adequately addressed, and I have no further comments on the scientific content. Only the following minor points remain before publication:

-The manuscript contains grammatical errors and awkward constructions in several places (e.g., L148, L358, L587–589, and similar instances throughout the text). A thorough language edit by a professional editing service or a fluent English speaker is recommended.

-The phrase "two latest LLMs" (Abstract, L217, L548) is no longer accurate, as newer models have since been released. A more time-robust wording such as "two widely used LLMs" is recommended.

-The image quality of Fig 2 is insufficient (blurry text with visible compression artifacts); a higher-resolution version should be provided.

Reviewer #2: The authors have satisfactorily addressed the comments raised during the previous review rounds, and the manuscript has been substantially improved. In particular, the methodological descriptions have been strengthened, the discussion has become more balanced, and the overly strong claims noted in the previous version have been largely moderated. The revisions have adequately addressed my previous concerns. I have no further comments and recommend the manuscript for publication in its current form.

**********

what does this mean?). If published, this will include your full peer review and any attached files.

If you choose “no”, your identity will remain anonymous but your review may still be made public.

**Do you want your identity to be public for this peer review?**  For information about this choice, including consent withdrawal, please see our Privacy Policy

Reviewer #1: No

Reviewer #2: No

**********

[NOTE: If reviewer comments were submitted as an attachment file, they will be attached to this email and accessible via the submission site. Please log into your account, locate the manuscript record, and check for the action link "View Attachments". If this link does not appear, there are no attachment files.]

To ensure your figures meet our technical requirements, please review our figure guidelines: https://journals.plos.org/plosone/s/figures

You may also use PLOS’s free figure tool, NAAS, to help you prepare publication quality figures: https://journals.plos.org/plosone/s/figures#loc-tools-for-figure-preparation.

NAAS will assess whether your figures meet our technical requirements by comparing each figure against our figure specifications.

# Author comment: Author response to Decision Letter 3

**DOI:** 10.1371/journal.pone.0358332.r006

12 Aug 2026

See the attached file "Response to Reviewer" (260806HumanAW_ResponsePone003toR1.docx). Reviewer 2 had no further comments in this round, so we made responses to only Reviewer 1.

Attachment Submitted filename: 260806HumanAW_ResponsePone003toR1.docx

# Editor report: Decision Letter 3

**DOI:** 10.1371/journal.pone.0358332.r007

**Authors:** Musa Aydin

1 Sep 2026

Attempts to investigate what words trivia experts focus on in quiz questions: Human-AI comparison through “LLM-as-a-judge” approach

PONE-D-25-63696R3

Dear Dr. Shirasuna,

We’re pleased to inform you that your manuscript has been judged scientifically suitable for publication and will be formally accepted for publication once it meets all outstanding technical requirements.

Within one week, you’ll receive an e-mail detailing the required amendments. When these have been addressed, you’ll receive a formal acceptance letter and your manuscript will be scheduled for publication.

An invoice will be generated when your article is formally accepted. Please note, if your institution has a publishing partnership with PLOS and your article meets the relevant criteria, all or part of your publication costs will be covered. Please make sure your user information is up-to-date by logging into Editorial Manager at Editorial Manager® and clicking the ‘Update My Information' link at the top of the page. For questions related to billing, please contact billing support.

If your institution or institutions have a press office, please notify them about your upcoming paper to help maximize its impact. If they’ll be preparing press materials, please inform our press team as soon as possible -- no later than 48 hours after receiving the formal acceptance. Your manuscript will remain under strict press embargo until 2 pm Eastern Time on the date of publication. For more information, please contact onepress@plos.org.

Kind regards,

Musa Aydin, Ph.D.

Academic Editor

PLOS One

Additional Editor Comments (optional):

Thank you for your careful revision and for addressing the comments raised during the previous review rounds. I have reviewed the revised manuscript and your responses, and I am satisfied that all remaining concerns have been adequately addressed. The manuscript has been substantially improved and is now suitable for publication.

I am therefore pleased to recommend acceptance for publication.

Reviewers' comments:

# Editor report: Acceptance letter

**DOI:** 10.1371/journal.pone.0358332.r008

**Authors:** Musa Aydin

PONE-D-25-63696R3

PLOS One

Dear Dr. Shirasuna,

I'm pleased to inform you that your manuscript has been deemed suitable for publication in PLOS One. Congratulations! Your manuscript is now being handed over to our production team.

At this stage, our production department will prepare your paper for publication. This includes ensuring the following:

* All references, tables, and figures are properly cited

* All relevant supporting information is included in the manuscript submission,

* There are no issues that prevent the paper from being properly typeset

You will receive further instructions from the production team, including instructions on how to review your proof when it is ready. Please keep in mind that we are working through a large volume of accepted articles, so please give us a few days to review your paper and let you know the next and final steps.

Lastly, if your institution or institutions have a press office, please let them know about your upcoming paper now to help maximize its impact. If they'll be preparing press materials, please inform our press team within the next 48 hours. Your manuscript will remain under strict press embargo until 2 pm Eastern Time on the date of publication. For more information, please contact onepress@plos.org.

You will receive an invoice from PLOS for your publication fee after your manuscript has reached the completed accept phase. If you receive an email requesting payment before acceptance or for any other service, this may be a phishing scheme. Learn how to identify phishing emails and protect your accounts at https://explore.plos.org/phishing.

If we can help with anything else, please email us at customercare@plos.org.

Thank you for submitting your work to PLOS One and supporting open access.

Kind regards,

PLOS One Editorial Office Staff

on behalf of

Assoc. Prof. Musa Aydin

Academic Editor

PLOS One
